# Small molecule inhibitors uncover synthetic genetic interactions of human flap endonuclease 1 (FEN1) with DNA damage response genes

**DOI:** 10.1371/journal.pone.0179278

**Published:** 2017-06-19

**Authors:** Thomas A. Ward, Peter J. McHugh, Stephen T. Durant

**Affiliations:** 1 AstraZeneca, Innovative Medicines and Early Development Biotech Unit, Oncology Bioscience, Alderley Park, Macclesfield, Cheshire, United Kingdom; 2 Department of Oncology, MRC Weatherall Institute of Molecular Medicine, John Radcliffe Hospital, University of Oxford, Oxford, United Kingdom; 3 AstraZeneca, Innovative Medicines and Early Development Biotech Unit, Oncology Bioscience, Little Chesterford, Cambridge, United Kingdom; The University of Hong Kong, HONG KONG

## Abstract

Flap endonuclease 1 (FEN1) is a structure selective endonuclease required for proficient DNA replication and the repair of DNA damage. Cellularly active inhibitors of this enzyme have previously been shown to induce a DNA damage response and, ultimately, cell death. High-throughput screens of human cancer cell-lines identify colorectal and gastric cell-lines with microsatellite instability (MSI) as enriched for cellular sensitivity to *N*-hydroxyurea series inhibitors of FEN1, but not the PARP inhibitor olaparib or other inhibitors of the DNA damage response. This sensitivity is due to a synthetic lethal interaction between *FEN1* and *MRE11A*, which is often mutated in MSI cancers through instabilities at a poly(T) microsatellite repeat. Disruption of *ATM* is similarly synthetic lethal with FEN1 inhibition, suggesting that disruption of FEN1 function leads to the accumulation of DNA double-strand breaks. These are likely a result of the accumulation of aberrant replication forks, that accumulate as a consequence of a failure in Okazaki fragment maturation, as inhibition of FEN1 is toxic in cells disrupted for the Fanconi anemia pathway and post-replication repair. Furthermore, RAD51 foci accumulate as a consequence of FEN1 inhibition and the toxicity of FEN1 inhibitors increases in cells disrupted for the homologous recombination pathway, suggesting a role for homologous recombination in the resolution of damage induced by FEN1 inhibition. Finally, FEN1 appears to be required for the repair of damage induced by olaparib and cisplatin within the Fanconi anemia pathway, and may play a role in the repair of damage associated with its own disruption.

## Introduction

Flap endonuclease 1 (FEN1) is a structure-specific endonuclease and prototypical member of the RAD2-superfamily [1–3], required for the removal of 5’ flaps that arise as a consequence of Okazaki fragment displacement by replicative polymerases during lagging strand synthesis [4, 5]. This process is critical for proficient and processive replication, with many cancer cells showing over-expression of *FEN1* [6–9]. Haploinsufficiency of *FEN1* is associated with abnormal cell-cycle progression and cancer predisposition with decreased survival, driven by an accumulation of replication-associated alterations in DNA, such as microsatellite instabilities (MSI) and tri-nucleotide repeat expansion [10–12]. FEN1 also plays a role in the maintenance of telomeres in the absence of telomerase [13], the processing of stalled replication forks [14, 15], and in a number of DNA damage repair processes, including base excision repair (BER) [16], alternative end-joining (alt-EJ) [17] and homologous recombination (HR) [18]. As a result, cells defective for FEN1 activity are sensitive to many DNA lesions [15, 19–24] and, therefore, FEN1 is an attractive target for drug discovery.

**Fig 1:**
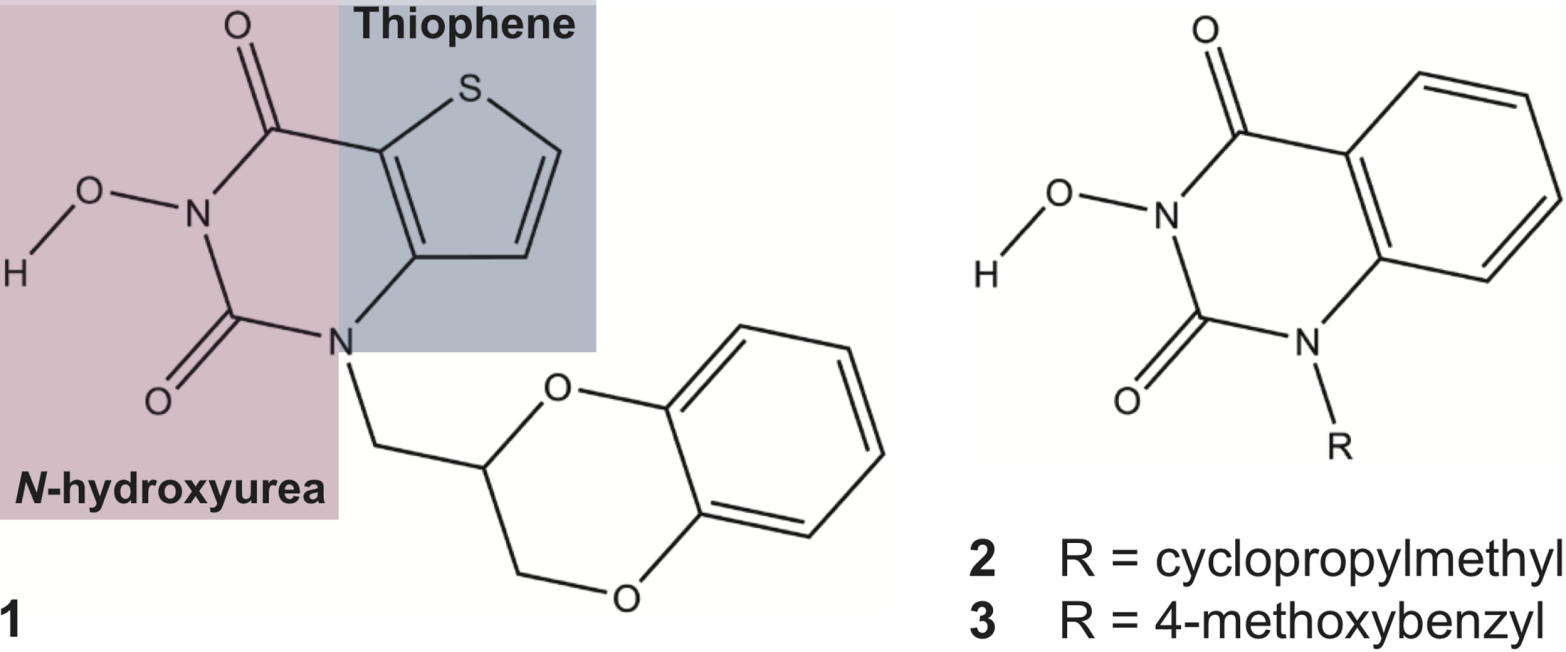
Chemical structure of *N-*hydroxyurea series inhibitors of FEN1 used in this study.

Previously it has been shown that the *N*-hydroxyurea series small molecule 1-(2,3-dihydro-1,4-benzodioxin-2-ylmethyl)-3-hydroxythieno[2,3-e]pyrimidine-2,4-dione (compound **1**; Fig 1) can inhibit FEN1 activity *in vitro* [25, 26]. We have shown that compound **1** co-crystallizes within the active site of FEN1 *in vitro*, with inhibition achieved partly through the co-ordination of Mg^2+^ ions by the *N*-hydroxyurea moiety of **1** (shaded purple in Fig 1) [25]. These metal ions are critical for base un-pairing; the disruption of base pairing within the substrate, close to the junction of single-strand to double-strand DNA, which induces an essential conformational change within the substrate duplex to allow for enzymatic cleavage [2, 27]. Further, the thiophene ring of **1** (shaded blue in Fig 1) sits within a hydrophobic pocket of FEN1 containing conserved active site residues Y40, D181 and R100 essential for substrate positioning [2, 27, 28]. Binding of **1** to the active site occurs in both a competitive and non-competitive manner with DNA, and, potentially, induces a dominant negative effect, with a FEN1-inhibitor ‘dead-end’ complex associating into replication and repair complexes, such as the PCNA-Pol∂-FEN1 complex that stimulates Okazaki fragment maturation [29, 30]. Further members of this *N*-hydroxyurea series (compounds **2**–**3**; Fig 1) have similarly been shown to inhibit FEN1 activity in biochemical assays [31]. This inhibitory activity is largely competitive with substrate, suggesting a different mode of action to **1** [25]. In the present study, we investigate further the therapeutic potential of *N*-hydroxyurea series inhibitors of FEN1.

**Fig 2:**
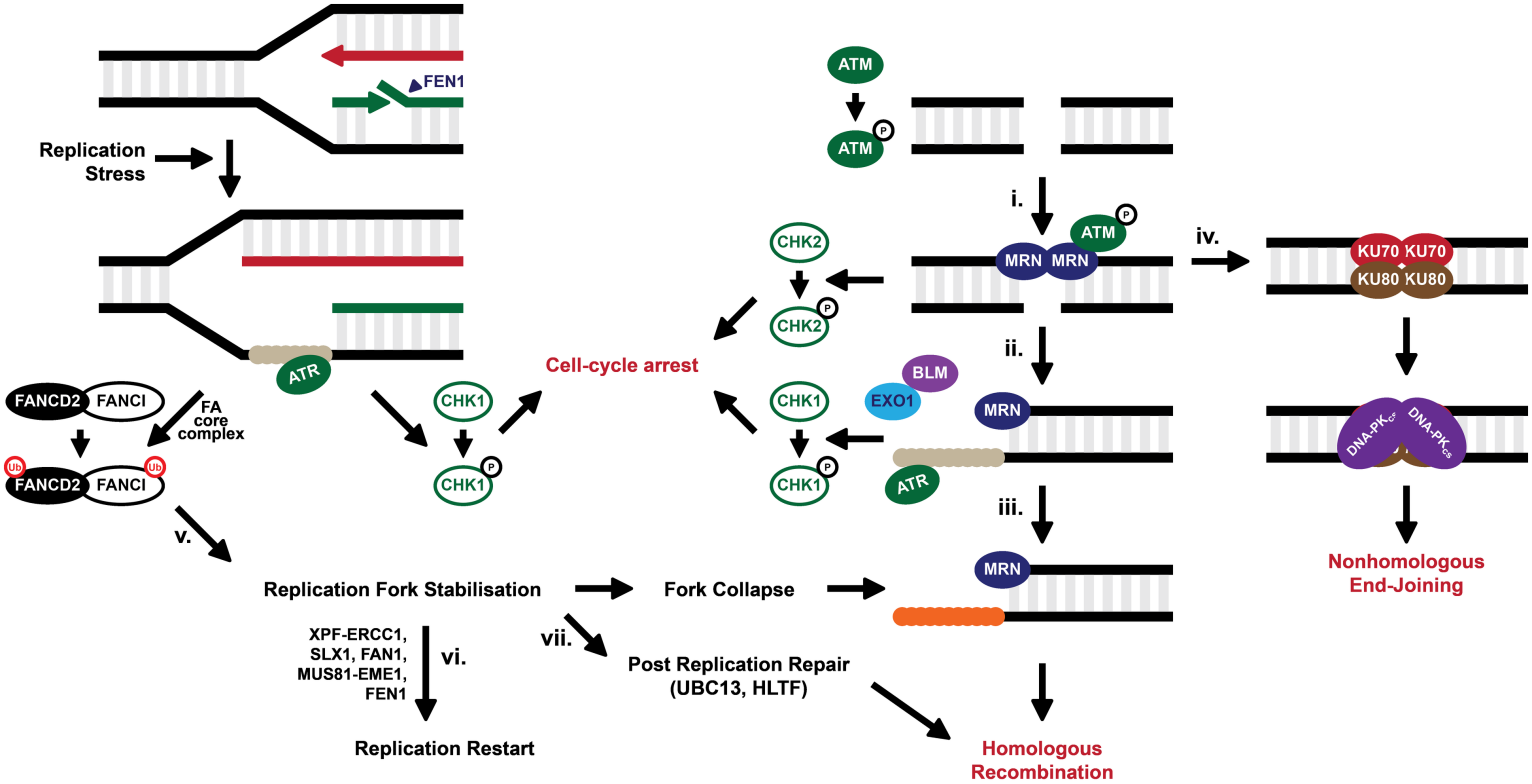
Model for the repair of DNA double-strand breaks. DSBs are recognised by the MRN complex (i.) which binds to the blunt DNA ends, holding them in close proximity. The exonuclease activity of MRE11A (or EXO1 in concert with BLM) is able to resect blunt ends, creating a 3’ ssDNA overhang, which becomes coated by the single-strand binding protein RPA (ii.), signalling for cell-cycle checkpoint arrest via ATR. RPA is displaced by RAD51 (iii.) to allow for HR. Alternatively, the MRN complex can be replaced by KU78/KU80 complex (iv.), protecting DNA ends from resection and promoting NHEJ through the binding of the DNA-PK catalytic subunit (DNA-PKcs). DSBs formed as a consequence of replication fork collapse require HR for their repair. Fork stalling, following replication stress for example, activates the FA pathway in an attempt to stabilise and protect the fork (v.). The FA core complex recognises the stalled fork and ubiquitinates the FANCD2-FANCI heterodimer. Stalled forks can be further processed by structure selective endonucleases to restore the replication fork (vi.) or cleave the fork to produce a DSB (vii.). Alternatively, the post-replication machinery can bypass damaged bases at stalled forks. One such pathway leads to template switching in a HR-mediated pathway.

Disruption or inhibition of FEN1 in both higher and lower eukaryotes induces a DNA damage response. *Saccharomyces cerevisiae* cells deficient for the *FEN1* homologue *RAD27* display temperature-dependent hyper-activation of post-replication repair (PRR) and DNA double-strand break (DSB) repair pathways following accumulation of unprocessed Okazaki fragments [19, 32, 33]. Previously [25] we demonstrated that *N-*hydroxyurea series molecules bind FEN1 protein *ex vivo* and that this binding translates to cellular activity, with mammalian cells treated with **1** initiating a DNA damage response in a dose-dependent manner. Similarly, we demonstrated that FEN1 inhibition activated the ATM checkpoint signalling pathway, the phosphorylation of histone H2AX and the ubiquitination of FANCD2 [25], suggesting the initiation of the Fanconi anemia (FA) pathway. The FA pathway is required for the stabilisation of stalled replication forks (Fig 2) and these data suggest that **1** induces replication-associated DNA damage. Moreover, it has been shown that inhibition of FEN1 is synthetic lethal with deficiencies in *MRE11A* [34], a member of the MRN (MRE11A-RAD50-NBS1) complex required for the sensing of DSBs and the activation of cell-cycle checkpoints (Fig 2) [35–38]. Following the sensing of DSBs by the MRN complex the kinases ATM and ATR induce DNA damage checkpoint signalling and repair is conducted via two main pathways: HR and non-homologous end-joining (NHEJ) (Fig 2). Previous data has suggested that mutation of *FEN1* in *Saccharomyces cerevisiae* and humans is synthetically lethal with HR factors [32, 39–41]. In the current study, we further investigate the activity of *N*-hydroxyurea series inhibitors of FEN1 in human cancer cells by high-throughput and targeted means. We identify synthetic genetic interactions between these inhibitors and disruption of DNA damage repair genes, and our data suggests FEN1 as a potential target for drug discovery in DNA repair-deficient cancers.

## Results and discussion

### A high-throughput screen identifies potential cancer sub-populations sensitive to chemical inhibitors of FEN1

**Fig 3:**
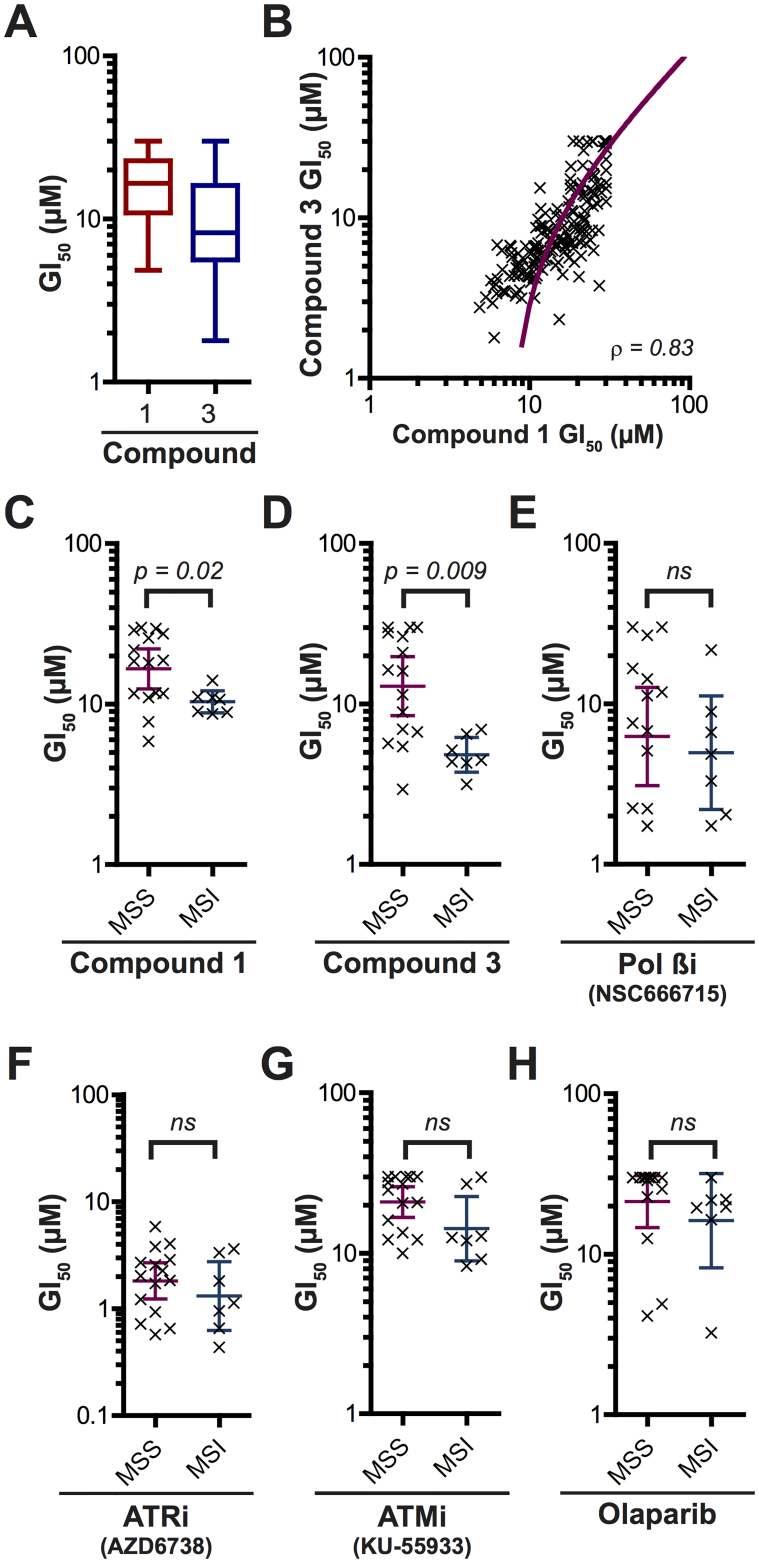
Cell-lines with MSI are specifically sensitive to chemical inhibition of FEN1 by compounds 1 and 3. **A**. Box and whisker diagram to show the variation in GI_50_ values for cell-lines subject to treatment with **1** and **3**. **B**. Correlation between the sensitivity of cell-lines to **1** and **3**. **C-H**. The influence of MSI on sensitivity to DNA repair inhibitors. ns = not significant * *p* < 0.05. ** *p* < 0.005.

Previously we have shown that chemical inhibition of FEN1 leads to DNA damage accumulation and ultimately cell death. We wanted to further examine the cytotoxic efficacy of FEN1 inhibition, to determine whether there was any tissue or genetic influence on those cells sensitive to treatment with these compounds. Initially, we conducted high-throughput screens across a panel of 280 cancer cell-lines derived from solid tumours from a number of tissue types. We continuously dosed each cell-line with 0–30 μM of three previously identified FEN1 inhibitors (compounds **1–3**) from the *N-*hydroxyurea series for 3 days and measured their growth inhibition effect (Table 1, S1 Table and Fig 3A). Dose-response results were obtained from 212 cell-lines treated with **1** with a mean GI_50_ of 15.5 μM. We were unable to calculate GI_50_ values for 26 of these cell-lines within the dose-range tested (i.e. the GI_50_ was greater than 30 μM), and these cell lines were deemed to be ‘resistant’ by this assay. *N*-hydroxyurea series compounds **2–3** similarly are inhibitors of FEN1 shown to have activity in biochemical assays [25, 26, 31]. FEN1 binding and inhibition by these molecules fit a competitive model while compound 1 fits a mixed non-competitive/competitive model, binding to FEN1 either in the presence or absence of DNA substrate [25]. We see little cellular activity for **2** in our high-throughput screen, with 79% cell-lines showing resistance to treatment with the dose-range specified. **3** is a structural relative of **2**, replacing a cyclopropylmethyl side-chain with a longer 4-methoxyphenyl. This modification enhanced cellular activity with a mean GI_50_ of 9.0 μM in the 195 cell-lines for which data was obtained, and 30 cell-lines (15%) showing resistance (Fig 3A). There was good Pearson’s correlation (ρ = 0.86) between sensitivity to **1** and **3** (Fig 3B), suggesting that cellular activity is likely due to the common inhibition of FEN1 despite the differing modes of inhibition.

**Table 1:** Summary of a high-throughput study to identify cell-lines sensitive to FEN1 inhibitors.

Compound	1	2	3
Cell-lines screened	212	180	195
Resistant cell-lines^a^	26 (12%)	143 (79%)	30 (15%)
Geometric mean	15.5	27.3	9.0
95% Confidence interval	1.0	0.7	1.2

^a^ Resistance was defined as GI_50_ greater than the maximum dose used in this study (30 μM).

We wanted to determine whether there was any tissue-type specificity to FEN1 inhibitor sensitivity. We therefore split the cell-lines into broad panels by tissue of origin and compared GI_50_ (Tables 2–4 and S1–S3 Figs). While cell-lines derived from head and neck cancers appear to be more resistant to treatment with **3** than other panels, there was otherwise very little difference between the mean GI_50_ across the tissue-types with any of the compounds tested. Subsequently, we further separated the cell-lines according to known cancer sub-types, with no significant difference in the mean GI_50_ of these cancer sub-types for any of the compounds tested (see S1–S3 Figs). Next we examined the sensitivity in cell-lines with mutations in genes associated with cancer (*KRAS*, *p53* and *PTEN*), again with no significant difference in sensitivity to FEN1 inhibition for all compounds tested (see S1–S3 Figs). Finally, we compared cell-lines with known MSI with cells shown to have stable microsatellites (MSS) from the same tissue-type (Table 5). MSI is a form of genetic hypermutability associated with variations in polynucleotide tracts. Approximately 10–15% of colorectal cancers (CRC) and ovarian cancers display MSI associated with a failure in genome surveillance by the mismatch repair (MMR) machinery, while MSI is also evident in head and neck, lung, oesophageal, pancreatic and prostate cancers [42–45]. Waterfall plots suggest that cell-lines with MSI were, largely, more sensitive to treatment with **1** and **3**, falling below the mean GI_50_ for all cell-lines tested (green bars, panel H in S1 Fig and panel H in S3 Fig) and showed a statistically significant increase in sensitivity compared with MSS cell-lines (Fig 3C and 3D). These data suggest MSI as a potential cancer subset with selective sensitivity to FEN1 inhibition. We considered that this effect may be through the inhibition of BER, a DNA repair pathway in which FEN1 plays a role. We therefore screened MSI and MSS cell-lines against another BER inhibitor, the cellular active DNA Polymerase β inhibitor NSC666715 [46, 47] (Fig 3E). No significant difference between the two cell populations was seen. Similarly, we screened these cell panels for sensitivity to inhibitors of other DNA damage response elements, including the ATR inhibitor AZD6738 [48], ATM inhibitor KU-55933 [49] or PARP inhibitor olaparib [50] (Fig 3F–3H). No significant difference in sensitivity to these inhibitors was seen between the two populations. These data would suggest that toxicity of compounds **1–3** in MSI cells is specific and not due to an addiction to the DNA damage response more generally.

**Table 2:** Tissue-specific sensitivity to compound 1. ^a^ Resistance was defined as GI_50_ greater than the maximum dose used in this study (30 μM).

Compound 1	Bladder	Breast	Colorectal	Gastric	Liver	Lung	Pancreatic	Prostate	Gynae	Head & Neck
Cell-lines screened	19	22	21	25	21	37	24	7	21	15
Resistant cell-lines^a^	0 (0%)	2 (9%)	4 (19%)	5 (20%)	3 (14%)	2 (5%)	3 (13%)	0 (0%)	7 (33%)	0 (0%)
Geometric mean^b^	13.6	13.1	16.7	15.0	20.1	12.1	16.5	17.1	23.1	20.0
95% Confidence interval	2.8	3.0	3.8	3.5	2.7	2.1	2.8	3.6	2.5	2.6

**Table 3:** Tissue-specific sensitivity to compound 2. ^a^ Resistance was defined as GI_50_ greater than the maximum dose used in this study (30 μM).

Compound 2	Bladder	Breast	Colorectal	Gastric	Liver	Lung	Pancreatic	Prostate	Gynae
Cell-lines screened	19	22	21	25	21	40	24	7	1
Resistant cell-lines^a^	18 (95%)	14 (64%)	15 (71%)	17 (68%)	19 (90%)	32 (80%)	21 (88%)	6 (86%)	1 (100%)
Geometric mean^b^	29.0	27.6	28.9	26.9	29.6	28.9	28.8	29.3	
95% Confidence interval	1.5	1.4	0.9	1.8	0.5	0.8	1.2	1.2	

**Table 4:** Tissue-specific sensitivity to compound 3. ^a^ Resistance was defined as GI_50_ greater than the maximum dose used in this study (30 μM).

Compound 3	Bladder	Breast	Colorectal	Gastric	Liver	Lung	Pancreatic	Prostate	Gynae	Head & Neck
Cell-lines screened	19	22	21	25	21	40	24	7	1	15
Resistant cell-lines^a^	1 (5%)	1 (5%)	5 (24%)	6 (24%)	4 (19%)	2 (5%)	2 (8%)	0 (0%)	0 (0%)	9 (60%)
Geometric mean^b^	8.2	6.4	11.0	11.5	11.0	7.4	8.1	9.2	5.2	19.8
95% Confidence interval	2.9	2.6	4.6	4.2	3.8	2.0	3.2	3.0		4.9

**Table 5:** *MRE11A* poly(T)_11_ mutations associated with MSI.

Cell-Line	MSI Status	MMR Defect	MRE11A poly(T)_11_ Intron 4 ^1^
HCT 116	MSI-H	MLH1	T10/T9
HCT-15/DLD-1	MSI-H	MSH6	T11/T9
LoVo	MSI-H	MSH2	T10/T10
RKO	MSI-H	MLH1	T10/T9/T8
SW48	MSI-H	MLH1	T10/T10
SNU-1	MSI-H	MLH1	-
SNU-638	MSI-H	MLH1	-
COLO 205	MSS	None	T11/T11
COLO 320DM	MSS	None	-
HT-29/WiDr	MSS	None	T11/T11
SK-CO-1	MSS	None	-
SW403	MSS	None	T11/T11
SW480	MSS	None	T11/T11
SW620	MSS	None	T11/T11
SW948	MSS	None	-
SNU-16	MSS	None	-
SNU-216	MSS	None	-
SNU-484	MSS	None	-
SNU-5	MSS	None	-
SNU-601	MSS	None	-
SNU-620	MSS	None	-
SNU-668	MSS	None	-
HCT116 CHR3	MSS	None^2^	T10/T9

^1^ Gene mutations taken from published data if known [51–53].

^2^ MLH1 is restored by the introduction of wild-type chromosome 3 and has been shown to be mismatch repair proficient.

### Colorectal cancer cell-lines with microsatellite instability-induced MRE11A deficiency are sensitised to down-regulation of FEN1

**Fig 4:**
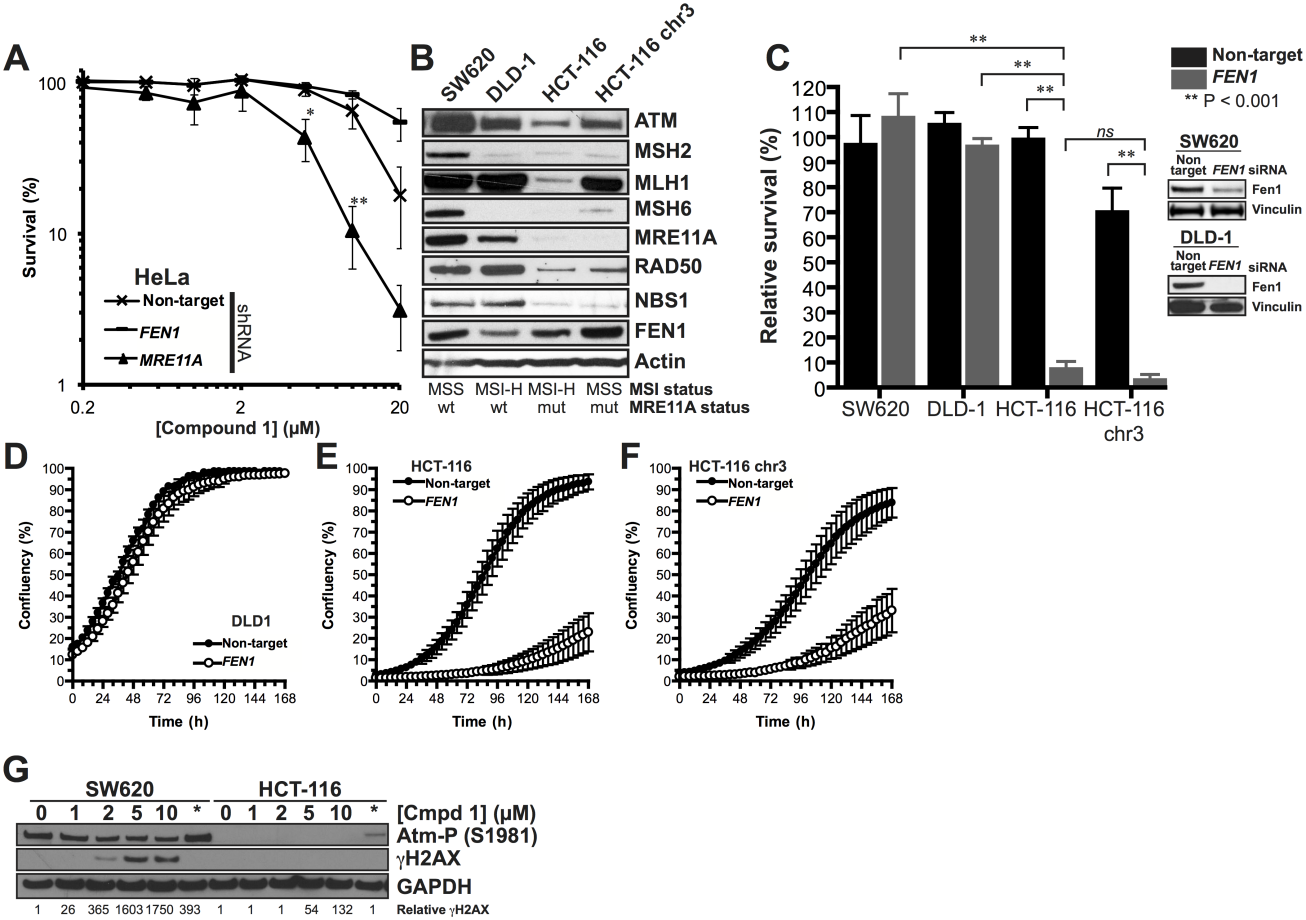
Sensitivity of MSI cell-lines to FEN1 inhibition is through *MRE11A* deficiency. **A**. Cells down-regulated for *MRE11A* by shRNA are sensitive to FEN1 inhibitor **1**. **B**. Confirmation that the MSI cell-line HCT-116 is disrupted for the MRN complex. Re-introduction of chromosome 3 (HCT-116 chr3) restores MLH1 without affecting MRE11A protein level. **C-F**. Down-regulation of FEN1 by siRNA is toxic in MSI cells devoid of MRN complex by clonogenic survival (**C**) or by measuring proliferation (**D-F**). Each data point is the mean of at least 3 individual repeats and the error bars represent the standard error. Significance was determined by student t-test. ns = not significant * *p* < 0.05. ** *p* < 0.005 **G**. HCT-116 cells fail to signal for the DNA damage response upon treatment with **1**.

DNA repair genes are frequently co-mutated with MMR due to polymorphisms at microsatellites resulting from an accumulation of replication errors. *MRE11A* for example is commonly mutated at a microsatellite comprising a tract of 11 thymine residues (poly(T)_11_ microsatellite) in intron 4 [51–54]. Previously, *MRE11A* has been shown to be synthetic lethal with *FEN1* [33, 35] and we therefore wanted to investigate the possibility that the selective sensitivity of FEN1 inhibitors is due to a loss of MRE11A. Of the seven confirmed MSI cell-lines examined in our high-throughput screen, five have been shown to have deleterious poly(T)_11_ mutations in one or more alleles of *MRE11A* (Table 5). We examined the role of MRE11A in the tolerance of **1** by initially treating cells stably expressing shRNA against *MRE11A* with the compound and compared sensitivity to an isogenic control cell-line expressing a non-targeting shRNA (Fig 4A). Cells disrupted for *MRE11A* were more sensitive to a dose escalation of **1**, and at 10 μM **1** we saw 11% survival in the *MRE11A* deficient cell-line compared to 66% in the control cell-line.

Next, we wanted to examine further whether inhibition of *FEN1* was responsible for lethality in *MRE11A* deficient MSI cells. We constructed a mini-panel of colorectal cancer cell-lines that differ by MSI-status and the mutation status of the *MRE11A* poly(T)_11_ microsatellite (Fig 4B), subject them to siRNA against *FEN1* and scored for clonogenic survival (Fig 4C) or ability to proliferate (Fig 4D–4F). SW620, an MSS cell-line [55], *MRE11A* expressing cell-line showed no sensitivity to *FEN1* disruption (Fig 4C & 4D). DLD-1 cells have MSI [55], yet these cells retain MRE11A protein as only one *MRE11A* poly(T)_11_ allele is mutated. Again, these cells are insensitive to *FEN1* siRNA treatment (Fig 4C). HCT-116 cells are similarly MSI [56], however, mutation of both *MRE11A* poly(T)_11_ alleles means this cell-line is devoid of MRE11A protein (Fig 4B). These cells are sensitive to *FEN1* siRNA treatment (Fig 4C & 4E), suggesting that disruption of FEN1 function by **1** is responsible for toxicity in this cell line. To examine the role played by MMR in sensitivity to FEN1 disruption, we also examined HCT-116 cells with chromosome 3 re-introduced, restoring *MLH1*. This has been shown to restore MMR function in these cells [57], without affecting MRE11A levels (Fig 4B). Both MMR-deficient and MMR-proficient cells were similarly sensitive to siRNA against *FEN1* (Fig 4C & 4F), suggesting that the toxicity is specific *MRE11A* defects and not MMR.

Our previous data suggested that **1** inhibits both FEN1 and EXO1 function *in vitro* but we were unable to determine whether this corresponded with cellular activity [26]. In an attempt to determine whether disruption of *EXO1* had an effect on the survival of *MRE11A-*deficient MSI cells, we also knocked-down *EXO1* in SW620 and HCT-116 cells and examined clonogenic potential. In both cases, disruption of *EXO1* had little effect on viability when compared to a non-target control (panel A in S4 Fig). Finally, we wanted to examine whether the disruption of *EXO1* played a role in sensitivity to **1**, explaining the severe and non-specific toxicity of **1** at high doses [26], we down-regulated *EXO1* by siRNA and subject the cells to increasing concentrations of **1** (panel B in S4 Fig). We find that cells disrupted for *EXO1* are no more sensitive to cells treated with a non-target control. This would suggest that the cytotoxicity induced by **1** is unlikely to be due to inhibition of EXO1 activity.

Finally, we wanted to examine the induction of DNA damage signalling pathways in these cells. Previously we have shown that treatment with **1** induces a DNA damage response [26]. In cells devoid of MRE11A, this signal is lost, with HCT-116 cells failing to accumulate γH2AX or activated ATM following treatment with **1** or olaparib (Fig 4G), suggesting that MRE11A is required for the activation of signalling pathways induced by damage associated with FEN1 or PARP inhibition.

### Cells devoid of the DNA double-strand break sensing machinery are sensitive to chemical inhibition of FEN1

**Fig 5:**
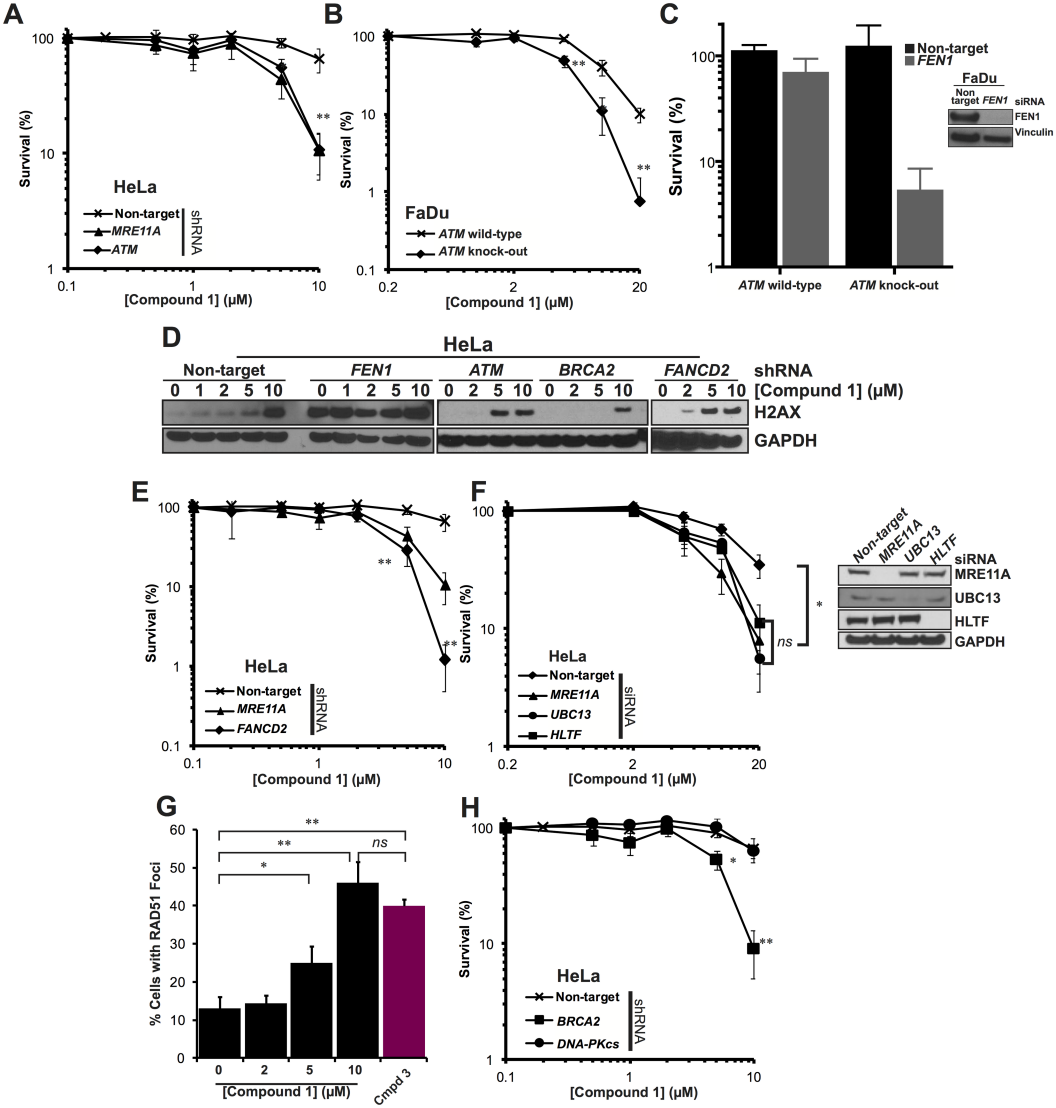
Cells disrupted for pathways required to maintain replication fork stability are sensitive to inhibition of FEN1. **A**. Clonogenic survival of cells stably expressing shRNA against *MRE11A*, *ATM* or a non-targeting control when treated with **1**. **B**. Clonogenic survival of FaDu cells or FaDu cells with all three ATM alleles knocked-out when treated with **1**. **C**. Clonogenic survival of FaDu cells or FaDu cells with all three ATM alleles knocked-out when treated with siRNA against FEN1. **D**. DNA damage response induced in cells expressing shRNA against *FEN1*, *ATM BRCA2*, *FANCD2* and a non-target control. **E-F**. Clonogenic survival of cells disrupted for *FANCD2* by shRNA (**E**) or genes required for PRR (*UBC13*, *HLTF*) by siRNA (**F**) compared to a non-target control. (**G**) Accumulation of Rad51 foci in cells treated with **1**. Data is collected from at least 500 cells per treatment. **H**. Clonogenic survival of cells disrupted for genes required for HR (*BRCA2*, *BLM*) and NHEJ (*DNA-PKcs*) by shRNA compared to a non-target control. In each clonogenic assay, data points represent the mean of at least 3 individual repeats and the error bars represent the standard error. Significance was determined by Student t-test. ns = not significant * *p* < 0.05. ** *p* < 0.005 **D**. DNA damage response induced in cells expressing shRNA against *FEN1*, *ATM* and a non-target control.

The MRN complex recognises DSBs and forms part of the signalling cascade that leads to damage induced cell-cycle arrest (Fig 2). This signalling cascade involves the activation of the kinase ATM by MRN [58]. ATM is activated when FEN1 is inhibited [26] and we hypothesised that cells deficient for ATM would likely be sensitive to **1**. Moreover, while the majority of MSI cell-lines identified above as sensitive to **1** are mutated for *MRE11A*, two have mutations in exon 6 of *ATM* resulting in a 22 bp deletion in exon 6 (SNU-1 and SNU-638) [59]. When we examined cells expressing shRNA against *ATM* for sensitivity to **1**, we found that the down-regulation of ATM lead to a potentiation of the toxic effects of FEN1 inhibition, phenocopying cells disrupted for *MRE11A* (Fig 5A). We further examined sensitivity in FaDu cells with all copies of *ATM* knocked out using the transcription activator-like effector nuclease (TALEN) system [60] with compound **1**. These cells also displayed greater toxicity than their parental cell-line (Fig 5B). Similarly, knock-down of *FEN1* by siRNA was toxic in these cells (Fig 5C), suggesting specificity in the activity of **1** in *ATM* deficient cells, further confirming synthetic lethality between *FEN1* and *ATM*.

Above we have shown that disruption of the MRN complex leads to both a loss in the activation of ATM and the accumulation of γH2AX. We examined **1**-induced γH2AX accumulation in cells disrupted for *ATM* by shRNA and found that, in contrast to *MRE11A* disruption, γH2AX accumulated more readily in *ATM* disrupted cells (Fig 5D). These data would suggest that the MRN complex activates ATM in response to FEN1 inhibition but ATM is not required for H2AX phosphorylation in this situation.

### A role for replication-coupled repair pathways in the tolerance of FEN1 inhibition

The MRN-ATM pathway has a role in the repair of DSBs repaired by NHEJ and canonical HR, such as those described in Fig 2i.–iv., and it is possible that the activation of this pathway in cells inhibited for FEN1 is due to an accumulation of such DNA breaks. However, this pathway has similarly been shown to be activated following the accumulation of more complex DNA secondary structures, such as stalled replication forks [61] and has been shown to promote replication restart following the collapse of the stalled forks into DSBs [62, 63]. Stalled replication forks are recognised by members of the FA pathway, which bind and stabilise the fork while co-ordinating downstream repair processes through monoubiquitinated FANCD2 (FANCD2-Ub) (Fig 2) [64–70]. An accumulation of ubiquitinated FANCD2 is therefore used as a marker for replication fork instability. Inhibition of FEN1, by treating with compound **1** or shRNA against *FEN1*, leads to an increase in FANCD2 mono-ubiquitination (FANCD2-Ub; panel F in S4 Fig and [26]), suggesting that inhibition of FEN1 leads to the accumulation of unstable replication forks. We hypothesised therefore that disruption of the FA pathway would lead to increased sensitivity to **1**. We obtained cells downregulated for *FANCD2* by shRNA and treated them with increasing doses of **1**. We find that, not only are these cells sensitive to **1**, this sensitivity is far greater than in any other cell-line examined (Fig 5E). At 10 μM **1**, 1% of *FANCD2* deficient cells survive compared with 66% in non-target control cells and approximately 10% in cells disrupted for *MRE11A* or *ATM*. Moreover, these cells accumulate DNA damage markers far more readily than in cells devoid of *ATM* (Fig 5D), suggesting that failure to stabilise stalled replication forks leads to increased DNA damage associate with **1** treatment. These data suggest that chemical inhibition of FEN1 leads to replication fork instabilities that, if left unrepaired, can lead to cell death.

In lower eukaryotes, disruption of *RAD27/FEN1* is synthetic lethal with disruption of the post-replication repair (PRR) damage tolerance pathway [34, 71]. PRR is required to overcome physical replication blocks or nicks in the DNA and is activated by regions of ssDNA (Fig 2) [72]. In the absence of *RA27*, PRR is required to prevent trinucleotide repeat expansion [73], which is often associated with FEN1 haploinsufficiency in vertebrates [13]. It has previously been demonstrated that ubiquitination of PCNA occurs following *RAD27* inactivation, a phenotype supressed by overexpression of *EXO1* [34]. Inactivation of *RAD27/FEN1* leads to an increase in length of 5’ flaps formed by replicative polymerases dissociating Okazaki fragments [74]. These longer flaps become coated in RPA and it has been postulated that these longer RPA-bound flaps signal for PRR [34]. Furthermore, disruption of *RAD27* leads to an accumulation of un-ligated single-strand nicks at Okazaki fragment [20, 33], which may also signal for PRR during subsequent rounds of replication. We wanted to examine this in our mammalian system so disrupted *UBC13* and the Rad5 homologue *HLTF* [75], which are PRR factors required for HR-mediated template switching [76–78], by siRNA and exposed cells to **1**. We found that disruption of either gene led to increased sensitivity to **1**, phenocopying the knock-down of *MRE11A* by the same method (Fig 5F). It is possible, therefore, that inhibition of FEN1 by this method leads to an accumulation of aberrant DNA replication structures on the lagging strand that engages PRR pathway for repair. In the absence of PRR, persistent replication fork instability ultimately results in cell death.

### A role for homologous recombination proteins in the repair of damage induced by FEN1 inhibition

Disruption of *RAD27* in lower eukaryotes is synthetically lethal with disruption of the HR pathway [32,39–40,79–80]. Since we have shown that inhibition of FEN1 largely recapitulates the phenotype seen in a yeast *rad27* cell, we wanted to examine the role played by HR factors in the tolerance of chemical inhibition of FEN1. HR is primed by the resection of DNA ends by MRE11A, resulting in a 3’ ssDNA overhang that rapidly becomes coated in RPA (Fig 2). RPA is displaced by RAD51 to form protein-DNA filaments that promote recombination. To determine whether HR is activated by **1**, we examined the accumulation of RAD51 foci post treatment, using the CellInsight high-content screening platform to count all cells containing defined RAD51 foci within the nucleus. We find that there is a dose-dependent increase in RAD51 foci following treatment with compound **1** (Fig 5G). The resulting foci are large, forming as a consequence of dynamic nuclear reorganisation post DNA damage [81, 82]. These foci are entirely nuclear (examples are shown in panel C of S4 Fig), however it is possible that they are foci prepared for sequestration to micronuclei, suggesting irreparable DNA damage [83]. This dose-dependent increase coincided with an accumulation of γH2AX foci (panel D of S4 Fig). Furthermore, when cells disrupted for *BRCA2* (which is required, among other DNA repair processes, for canonical HR) by shRNA were subject to increasing concentrations of **1**, we found increased sensitivity that phenocopied *MRE11A* and *ATM* disruption (Fig 5H). Disruption of BRCA2, however, did not increase γH2AX accumulation (Fig 5D), suggesting a role for BRCA2 downstream of MRE11A. Cells disrupted for *DNA-PKcs*, a gene required for the non-homologous end-joining (NHEJ)–a pathway utilised frequently for the repair of simple DSBs that supresses HR (see also Fig 2)–were no more sensitive to **1** than a non-target control.

Above we have shown that disruption of DNA damage repair proteins contributes to cellular sensitivity to FEN1 inhibition. We therefore re-examined our high-throughput cell killing data set to determine whether gene expression and copy number influence sensitivity to our compounds. We found that cellular sensitivity to *N*-hydroxyurea series compounds did not correlate with expression of *FANCD2*, *MRE11A*, *ATM* or *BRCA2* with any statistical significance (S6 Fig).

### FEN1 is epistatic with FANCD2 for the repair of olaparib- and cisplatin-induced DNA damage

**Fig 6:**
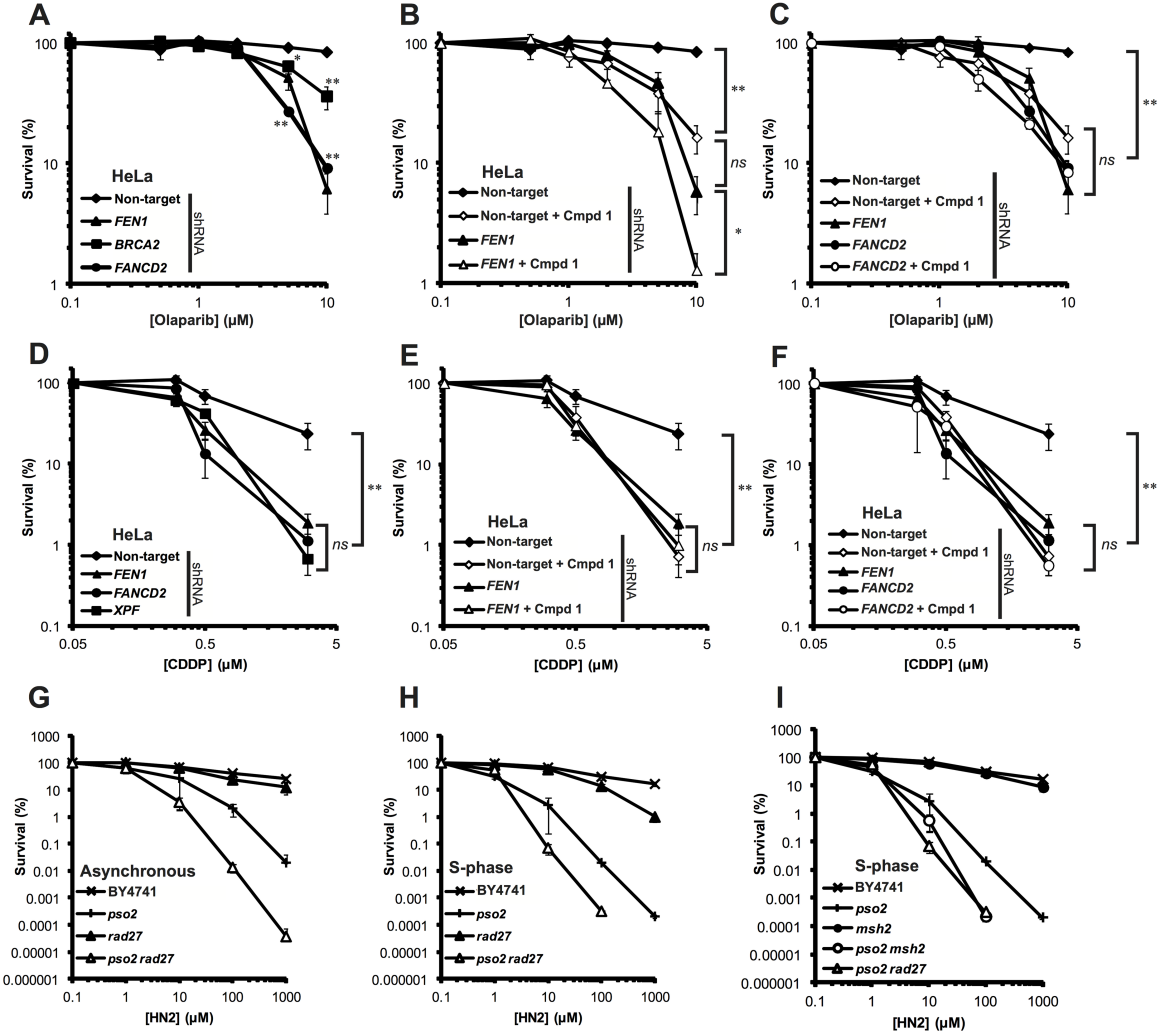
*FEN1* is epistatic with *FANCD2* for the repair of olaparib- and cisplatin-induced DNA damage. **A**. **Olaparib** sensitivity of cells disrupted for *FEN1*, *FANCD2* or *BRCA2* by shRNA compared to a non-target control. **B**. Sensitisation of cells to olaparib following treatment with 10 μM **1**. **C**. Epistasis analysis of FEN1 inhibition and *FANCD2* depletion following exposure to olaparib. **D**. **Cisplatin** sensitivity of cells disrupted for *FEN1*, *XPF* and *FANCD2* by shRNA compared to a non-target control. **E**. Sensitisation of cells to cisplatin following treatment with 5 μM **1**. **F**. Epistasis analysis of FEN1 inhibition and *FANCD2* depletion following exposure to cisplatin. **G-I**. Sensitivity of *Saccharomyces cerevisiae* strains deleted for *rad27*, *pso2*, *msh2* singularly and in combination either in asynchronous culture (**G**) or synchronised in S-phase (**H-I**). In all cases, each data point is the mean of at least 3 individual repeats and the error bars represent the standard error. Significance was determined by student t-test. ns = not significant * *p* < 0.05. ** *p* < 0.005.

FEN1 has been implicated in the repair of damage induced by PARP inhibitors [19, 84]. Such inhibitors both prevent the repair of alkylated bases through inhibition of BER or the trapping of PARP1/2 to DNA breaks. Such lesions stall replication forks and, if left unrepair, are converted to DSBs following fork collapse. Disruption of the DSB recognition machinery, HR or the FA pathways sensitise cells to PARP inhibitors, such as olaparib or veliparib [53, 79, 80, 84–88]. Since the data above suggests a synthetic lethal interaction between FEN1 inhibition and the disruption of genes known to be required for the repair of damage induced by PARP inhibitors, we wanted to examine whether FEN1 inhibition would have an effect on olaparib sensitivity in cells disrupted for these pathways, particularly the FA pathway and *BRCA2*. Consistent with previous data, we found that cells down-regulated for *FEN1*, *FANCD2* or *BRCA2* with shRNA were profoundly more sensitive to olaparib than non-target control cells (Fig 6A). Next we examined whether treatment with **1** was able to recapitulate this sensitivity in cells otherwise wild-type for *FEN1*. We found that, treatment with 10 μM **1** sensitised cells to a similar extent to *FEN1* knockdown by shRNA (panel A in S5 Fig and Fig 6B). Interestingly, cells disrupted for *FEN1* by shRNA were sensitised to olaparib further by treatment with **1**. This could be a result of the inactivation of remaining FEN1, the inactivation of EXO1 or through a dominant negative effect. To determine whether FEN1 inhibition would potentiate the toxic effects of olaparib in *BRCA2* and *FANCD2* deficient cells, we pre-treated cells expressing shRNA against either gene with 10 μM **1** before adding varying concentrations of olaparib. While *BRCA2* disrupted cells showed intermediate sensitivity to olaparib, co-treatment with **1** led to an increase in sensitivity that phenocopied a *FEN1* disrupted cell-line (panel B of S5 Fig). Similarly, *FANCD2* disrupted cells treated with **1** were no more sensitive to olaparib than *FANCD2* cells treated with DMSO alone (Fig 6C). These data would suggest that FEN1 is epistatic with BRCA2 and the FA pathway for the repair of olaparib-induced DNA damage, while it was shown above that disruption of FEN1 induces damage that requires the FA pathway for its repair. To examine whether this epistasis was specific for olaparib or for other replication-coupled repair, we repeated these experiments replacing olaparib with the cross-linking agent cisplatin (CDDP). The *Saccharomyces cerevisiae* homologue of FEN1, Rad27, plays a role in the repair of DNA interstrand cross-links (ICLs) in a pathway independent of the 5’-3’ exonuclease Pso2 [22, 25]. Similarly, FEN1 has been implicated in the repair of cisplatin (CDDP)-induced ICLs [8], however no formal role for FEN1 has been assigned in this pathway. We treated cells disrupted for *FEN1* by shRNA with CDDP and compared sensitivity to cells disrupted for known ICL-repair genes (*FANCD2* and *XPF*) (Fig 6D). We found that, not only were *FEN1* disrupted cells sensitive to CDDP, this sensitivity phenocopied cells disrupted for *FANCD2* and *XPF*. Pre-treatment with **1**, again, sensitised *FEN1* proficient cells to a level consistent with inhibition of the enzyme (Fig 6E) and was epistatic with down-regulation of *FANCD2* (Fig 6F). These data, together with the data described above, suggest a role for FEN1 in the FA pathway for the repair of replication-associated DNA damage. Since *S*. *cerevisiae* has a prototypical FA pathway that functions in the absence of Pso2 [89–92], we wanted to examine whether this putative role for FEN1 was conserved. *S*. *cerevisiae RAD27* was deleted in a *pso2* background and the resulting strains treated with nitrogen mustard (HN2) in asynchronous culture or cultures held in S-phase. Consistent with previous data [22, 25], we found that *rad27* cells were insensitive to HN2 in asynchronous culture, yet co-disruption of *pso2* led to a synergistic increase in sensitivity (Fig 6G). This phenotype persisted in cells held in S-phase (Fig 6H) with *pso2 rad27* phenocopying *pso2 msh2* (Fig 6I), suggesting a conserved role for Rad27/FEN1 in the *Saccharomyces cerevisiae* FA pathway. These data together hint at a role for FEN1 in a FA-related pathway. This is somewhat consistent with previous data as FEN1 activity has been shown to be stimulated by the FA core-complex member FANCA [93], and has been shown to be involved in replication fork processing and restart [15, 94]. Collectively, our data suggest that FEN1 inhibition results in accumulation of DNA damage, which requires the FA pathway for repair. This damage is most likely associated with a failure to process 5’ flaps created during lagging-strand synthesis, leading to replication fork instability and, ultimately, collapse into a DSB. Given that FEN1 appears to have a role in the FA pathway for the repair of olaparib or CDDP-induced DNA damage, it is likely that FEN1 itself would be required for the repair of damage induced by chemical inhibition of FEN1.

### A model for the induction and repair of DNA damage associated with chemical inhibition of FEN1

**Fig 7:**
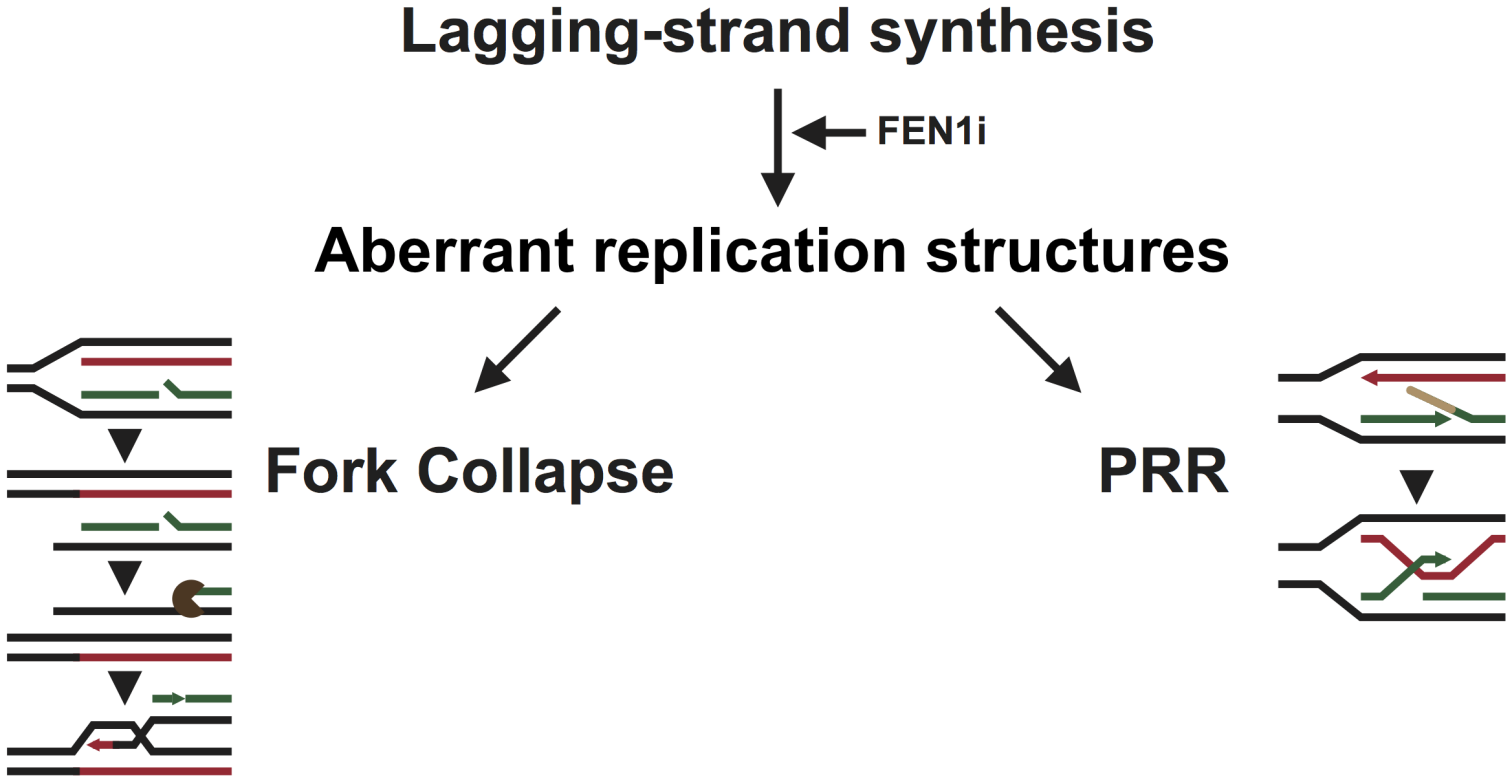
Current model for the formation and repair of DNA damage following FEN1 inhibition. The inhibition of FEN1 leads to the accumulation of immature Okazaki fragments bound by RPA, accumulating aberrant replication structures that destabilise the replication fork. The PRR machinery is thought to allow for the tolerance of such structures by switching template strand, however failure to do so in a timely manner could lead to the stalling and, ultimately, collapse of the fork. The broken fork would require processing by endonucleases to create a 3’ overhang able to invade into back into the dsDNA and re-start replication. Persistent inhibition of FEN1 would ultimately lead to an overwhelming level of DNA damage and, ultimately, cell death.

The data presented above shows that inhibition of FEN1 by *N*-hydroxyurea series inhibitors leads to specific cell killing in cells disrupted for a number of DNA damage pathways. These inhibitors activate the FA pathway (S4 Fig and [26]), suggesting an accumulation of aberrant replication structures following FEN1 inhibition (Fig 7). Failure to stabilise and resolve these structures appears to be extremely toxic as disruption of FANCD2 leads to extreme sensitivity to FEN1 inhibition (Fig 5E). It is currently unclear what the precise nature of these aberrant structures, however we have genetic evidence to suggest that they are generated behind the replication fork, since disruption of error-free PRR leads to increased sensitivity to FEN1 inhibitors (Fig 5F). In yeast cells inhibited for Okazaki fragment maturation, PRR is thought to be activated in response to the accumulation of long 5’ flaps [34], nicks [71] and abnormal secondary structures in the DNA such as hairpins [13, 73], and it is possible that these structures are generated by FEN1 inhibitors.

BRCA2 and RAD51 are proteins with canonical roles in HR but they also play a role in the protection of forks against degradation [63, 95]. Both proteins are required in the absence of functional FEN1 (Fig 5G and 5H). It is possible that the role these proteins play in the tolerance of FEN1 inhibitors is solely in the protection of stalled replication forks, but it is equally possible that canonical HR is activated in the absence of FEN1 [33, 40–42]. Failure to repair replication forks leads to collapse and the generation of DSBs. These DSBs are repaired by non-reciprocal HR, in which the broken arm of the fork is processed by MRN complex to create a 3’overhang, which ultimately invades into the complete dsDNA to re-establish the replication fork. We and others [35] have shown that MRE11A is required in the absence of FEN1 activity (Fig 4) and here we have shown that cells with MSI that display disruption in MRN complex show selective sensitivity to FEN1 inhibitors (Fig 3). MRE11A and ATM act in the same pathway for the repair of DSBs (Fig 2) [96] and are both required for the repair of damage induced by FEN1 inhibition (Fig 5A–5D). ATM is activated by FEN1 inhibitors [26] in an MRE11A dependent manner (Fig 4G) suggesting the presence of DSBs. There is, however, no requirement for NHEJ in the repair of damage induced by FEN1 inhibitors (Fig 5H), suggesting that the damage induced is not a simple two-ended DSB. MRE11A, however, also plays a role in the processing and restart of stalled replication forks [63, 97] and it may be that the function of MRE11A is in the processing of aberrant replication structures.

Finally, we have shown that FEN1 operates in the repair of lesions induced by cisplatin, nitrogen mustard and olaparib in a pathway that also requires FANCD2 (Fig 6). This suggests a role for FEN1 in replication-coupled DNA repair, which could include damage induced by FEN1 inhibition. The role for FEN1 in the repair of these lesions is unclear, however there is evidence to suggest that FEN1 plays a role in replication restart and the processing of stalled replication forks [15, 16, 94, 98]. It would be interesting to determine the precise function of FEN1 in this pathway.

The work presented here suggests that inhibitors of FEN1 would be effective in targeting cells deleted, mutated or disrupted for a number of DNA damage response genes, many of which are associated with cancer [99]. Similarly, FEN1 inhibitors sensitise cells to chemotherapeutics such as cisplatin, and DNA repair inhibitors such as olaparib. The work presented here supports FEN1 as a possible target for oncology drug discovery.

## Materials and methods

### High-throughput cell-killing assay

Toxicity was calculated by using a modified, high-throughput version of the MTS assay. Cells were plated at an appropriate density in 96-well plates and allowed to adhere for 24 h. Compound, diluted in DMSO, was added to a final concentration of between 0 and 30 μM and plates were incubated at 37°C for 0 or 2 days. Subsequently, CellTiter96 AQ_ueous_ MTS (Sigma) was added as per the manufacturers recommendations and plates were incubated at 37°C for 2 h. Absorbance was measured using a Tecan Ultra microplate reader (Tecan Life Sciences) and data validated and analysed as per internal data handling methodology.

### Cell-lines and media

HeLa, FaDu, SW-620, DLD1, HCT-116 and HCT-116 chr3 were all obtained from internal stocks. The FaDu *ATM* triple knock-out cell-line was created using transcription activator-like effector nucleases (TALEN) by AstraZeneca (Discovery Sciences, Sweden). SilenciX cell-lines were all obtained from Tebu Bioscience and knockdown of the stated gene was confirmed externally by qPCR. All cell-lines were grown in DMEM (Sigma Aldrich) media. SilenciX cell-lines were maintained in DMEM media supplemented with 125 μg/mL hygromycin B to prevent the loss of shRNA-expression plasmids. All cell-lines were verified in-house by short tandem repeat (STR) fingerprinting and routinely tested for mycoplasma.

### siRNA knockdown

siRNA-lipofectamine complexes were created using Lipofectamine RNAiMAX (Thermo Fisher Scientific) as per the manufacturer’s instructions. All siRNAs used in this study were Dharmacon ON-TARGETplus SMARTpools (GE Healthcare). siRNA was added at a final concentration of 20 nM and allowed to grow for 24 hours. Cells were subsequently washed and fresh media applied to avoid lipofectamine toxicity. For knock-down confirmation, cells were trypsinised after 3 days in fresh media, collected and lysed in Cell Panel Lysis Buffer (5 mM Tris-HCl, 3 mM EDTA, 3 mM EGTA, 50 mM NaF, 2 mM sodium orthovanadate, 0.27 M sucrose, 10 mM ß-glycerophosphate, 5 mM sodium pyrophosphate, and 0.5% Triton X-100) supplemented with complete protease and phosSTOP phosphotase inhibitors (both Roche). Examples of knock-down efficiency are shown with the figures. These cells were subsequently used for down-stream experiments discussed below.

### Mammalian colony formation assay

Cells were plated at an appropriate density and allowed to settle for >24 hours in appropriate media. Media was supplemented with compounds (**1**, olaparib, cisplatin) at the stated doses and allowed to grow until colonies of >50 cells were achieved. For combination experiments, growth media was changed after 24 hours for media supplemented with 5 or 10 μM **1** (stated in the Fig legend) and cells were allowed to grow for 24 hours. Subsequently, media was further supplemented with drug at the stated dose and allowed to grow until colonies of >50 cells were achieved. For ionising radiation experiments, cells were seeded at the stated dose and allowed to settle for 24 hours. Media was supplemented with 5 μM **1** and allowed to grow for 24 hours. Cells were subsequently exposed to ionising radiation at the stated dose. For siRNA knockdown toxicity experiments, cells were treated with siRNA as described above and allowed to grow in fresh media until colonies of >50 cells were achieved.

### MRN complex and MMR status determination

SW620, DLD1, HCT-116 and HCT-116 chr3 cells were trypsinised, collected and lysed in Cell Panel Lysis Buffer with complete protease and phosphotase inhibitors. Proteins were separated by gel electrophoresis and transferred to nitrocellulose membrane by Western blot. Membranes were probed, at a concentration of 1:1000 unless stated otherwise, for ATM (sc-23921, Santa Cruz Biotechnology), MSH2 (556349, BD Pharmigen), MLH1 (WH0004292M2, Sigma Aldrich), MSH6 (610918, BD Transduction), MRE11A (ab214, Abcam), RAD50 (611010, BD Transduction), NBS1 (NB100-143, Novus Biologicals), FEN1 (ab109132, Abcam) and β-Actin (A5441, Sigma Aldrich).

### Damage induction assay

DNA damage induction was determined as described previously [26]. Briefly, cells were plated at an appropriate density and allowed to settle overnight. Media was supplemented with **1** at the dose stated and cells were allowed to grow for 4 days with constant exposure. Cells were trypsinised, collected and lysed in Cell Panel Lysis Buffer with complete protease and phosphotase inhibitors. Proteins were separated by gel electrophoresis and transferred to nitrocellulose membrane by Western blot. Membranes were probed, at a concentration of 1:1000 unless stated otherwise, for γH2AX (#2577, Cell Signaling Technology; 1:500), GAPDH (#3683, Cell Signaling Technology; 1:5000), phospho-ATM (Ser1981) (ab81292, Abcam) and FANCD2 (sc-20022, Santa Cruz Biotechnology).

### Immunofluorescence

Cells were seeded in 96-well plates at an appropriate density and allowed to settle for 24 hours. Media was supplemented with compound at the stated dose and allowed to grow with constant dosing for 4 days. Cells were fixed in a final concentration of 4% paraformaldehyde for 20 min and washed 3 times in PBSA. Cells were permeablized for 4 min in 0.5% triton X-100 in PBSA, washed and probed with anti-γH2AX (JBW301, Millipore) or anti-RAD51 (ABE257, Millipore) at 1:500 for 48 hours at 4°C. Cells were washed in 0.05% Tween in PBSA 3 times for 5 min before probing for 1 hour at room temperature with AlexaFluor 488 or AlexaFlour 546-conjugated secondary antibodies (both Life Technologies) at a concentration of 1:500, and Hoechst DNA stain at 1:2000. Immunoflourescence was examined using CellInsight CX5 (Thermo Fisher) and foci were quantified using the software and algorithms provided to specifically count foci formed within the nucleus. Example images of foci are shown in panels C and E of S4 Fig.

### Yeast strains

Strains used in this study (S2 Table) were created as previously described [89]. Gene deletion was carried out by micro-homology targeted gene disruption using the pFA6a vector series and its derivatives [100–102] or generated by synthetic genetic analysis methodology [103]. Deletions were confirmed by PCR analysis and restriction enzyme digestion. Cells were grown in YPD media and plated onto YPD agar.

### Yeast nitrogen mustard sensitivity

Nitrogen mustard sensitivity was determined as previously described [25, 89]. Exponentially growing cells were treated with increasing doses of nitrogen mustard in PBSA for 1 h at 30°C. Cells were washed and plated at an appropriate density and colonies were scored after 3 days incubation at 30°C. Percent survival is calculated as a fraction of an untreated control.

### Replication-dependent nitrogen mustard sensitivity

Replication-dependent sensitivity was determined as previously described [25]. Cells were synchronised in G_1_-phase of the cell-cycle with alpha mating factor for 2 h before being washed in PBSA and re-suspended in fresh YPD. Cells allowed to enter S-phase for 10 min before being treated with nitrogen mustard as described above. In pilot experiments, cell-cycle synchronisation was conducted by FACS.

## Supporting information

S1 Fig High-throughput screen for genetic backgrounds sensitive to 1. Scatter plots show cell-line sensitivities to treatment with **1**, broadly grouped by tissue of origin (**A**), tissue sub-group (**B-D**) or oncogene mutation status (**E-G**). Waterfall plot (**H**) shows all cell-line sensitivities to **1**, with those confirmed to be MSI highlighted in green.
(TIF)

S2 Fig High-throughput screen for genetic backgrounds sensitive to 2. Scatter plots show cell-line sensitivities to treatment with **2**, broadly grouped by tissue of origin (**A**), tissue sub-group (**B-D**) or oncogene mutation status (**E-G**). Waterfall plot (**H**) shows all cell-line sensitivities to **2**, with those confirmed to be MSI highlighted in green.
(TIF)

S3 Fig High-throughput screen for genetic backgrounds sensitive to 3. Scatter plots show cell-line sensitivities to treatment with **3**, broadly grouped by tissue of origin (**A**), tissue sub-group (**B-D**) or oncogene mutation status (**E-G**). Waterfall plot (**H**) shows all cell-line sensitivities to **3**, with those confirmed to be MSI highlighted in green.
(TIF)

S4 Fig **A**. Clonogenic survival of SW620 and HCT-116 cells treated with siRNA against *EXO1*. **B**. Clonogeneic survival of HeLa cells disrupted for *EXO1* and *MRE11* following treatment with **1**. **C**. Example of γH2AX foci induced by **1** or olaparib. **D**. Dose dependent increase in cells with at least 5 γH2AX foci. Data is a quantification from at least 500 cells. **E**. Example of RAD51 foci induced by **1** or **3**. **G**. Activation of the Fanconi anemia pathway in cells disrupted for *FEN1* compared to a non-target control.
(TIF)

S5 Fig **A**. Effect treatment with 5 μM and 10 μM **1** has on sensitivity to olaparib in cells with wild-type levels of *FEN1*. **B**. Epistasis analysis of FEN1 inhibition and *BRCA2* depletion following exposure to olaparib.
(TIF)

S6 Fig There is no correlation between expression of *FANCD2* (A), *MRE11A* (B), *ATM* (C) or *BRCA2* (D) and sensitivity to compound 1 (left), 2 (middle) or 3 (right). *ρ* denotes the Pearson’s correlation coefficient.
(TIF)

S7 Fig Original Western blots used in the construction of panel B of Fig 4. (TIF)

S8 Fig Original Western blots used in the construction of panel C of Fig 4. (TIF)

S9 Fig Original Western blots used in the construction of panel G of Fig 4. (TIF)

S10 Fig Original Western blots used in the construction of panel C in Fig 5. (TIF)

S11 Fig Original Western blots used in the construction of panel D in Fig 5. (TIF)

S12 Fig Original Western blots used in the construction of panel F in Fig 5. (TIF)

S13 Fig Original Western blots used in the construction of panel A in S4 Fig. (TIF)

S14 Fig Original Western blots used in the construction of panel B in S4 Fig. (TIF)

S15 Fig Original Western blots used in the construction of panel F in S4 Fig. (TIF)

S1 Table High-throughput screen for genetic backgrounds sensitive to *N*-hydroxyurea series inhibitors of FEN1 –Raw GI_50_ values. (DOCX)

S2 Table Yeast strains used in this study. * Strain kindly donated by the Boone Lab. ^#^ Strains purchased from Open Biosystems.
(DOCX)
