# Supplementary figures and images for: Small molecule inhibitors uncover synthetic genetic interactions of human flap endonuclease 1 (FEN1) with DNA damage response genes
Source: PLoS One. 2017 Jun 19;12(6):e0179278. doi: 10.1371/journal.pone.0179278 (PMC5476263; doi:10.1371/journal.pone.0179278)

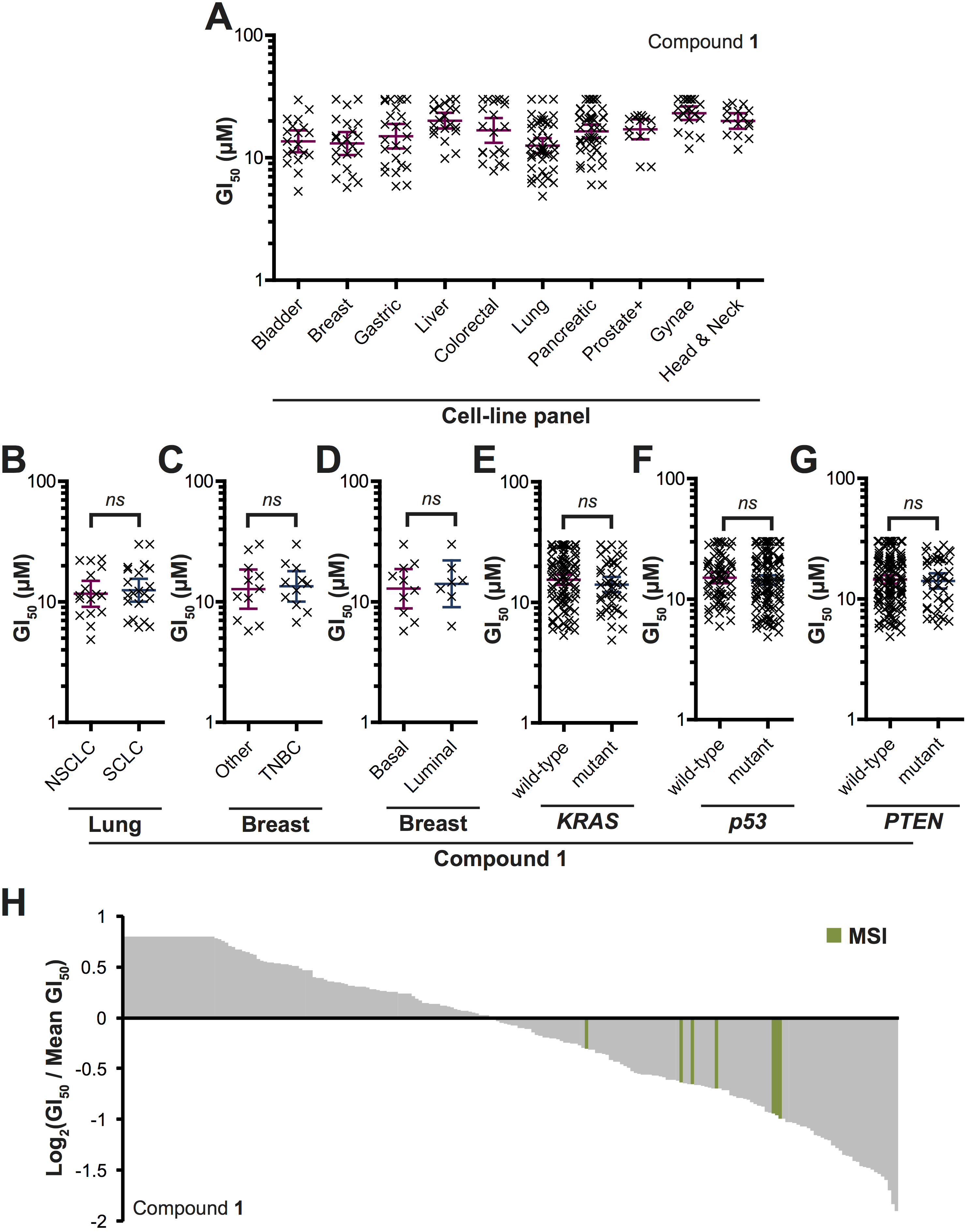

Supplement: S1 Fig — Scatter plots show cell-line sensitivities to treatment with 1, broadly grouped by tissue of origin (A), tissue sub-group (B-D) or oncogene mutation status (E-G). Waterfall plot (H) shows all cell-line sensitivities to 1, with those confirmed to be MSI highlighted in green. (TIF) [file pone.0179278.s001.tif]

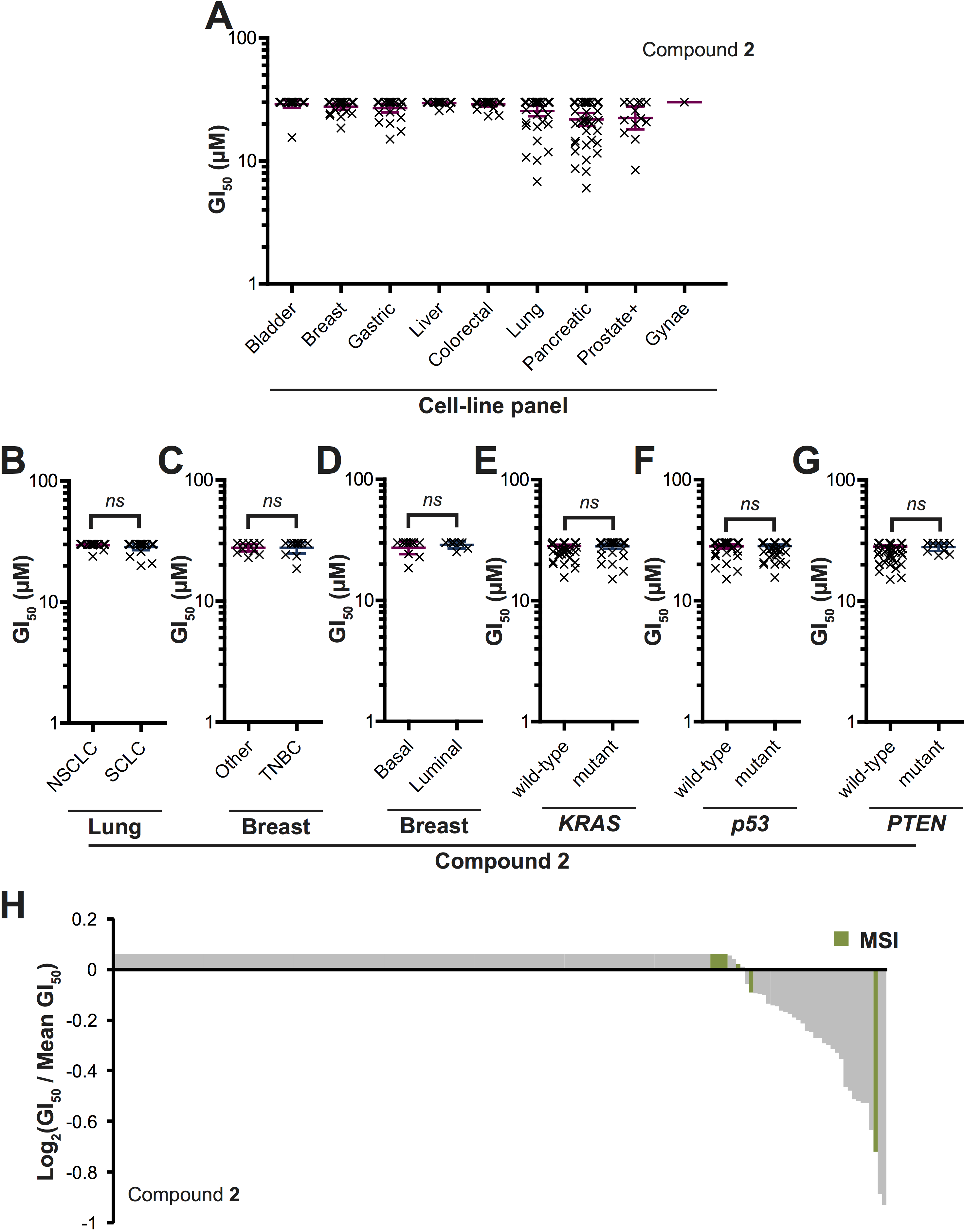

Supplement: S2 Fig — Scatter plots show cell-line sensitivities to treatment with 2, broadly grouped by tissue of origin (A), tissue sub-group (B-D) or oncogene mutation status (E-G). Waterfall plot (H) shows all cell-line sensitivities to 2, with those confirmed to be MSI highlighted in green. (TIF) [file pone.0179278.s002.tif]

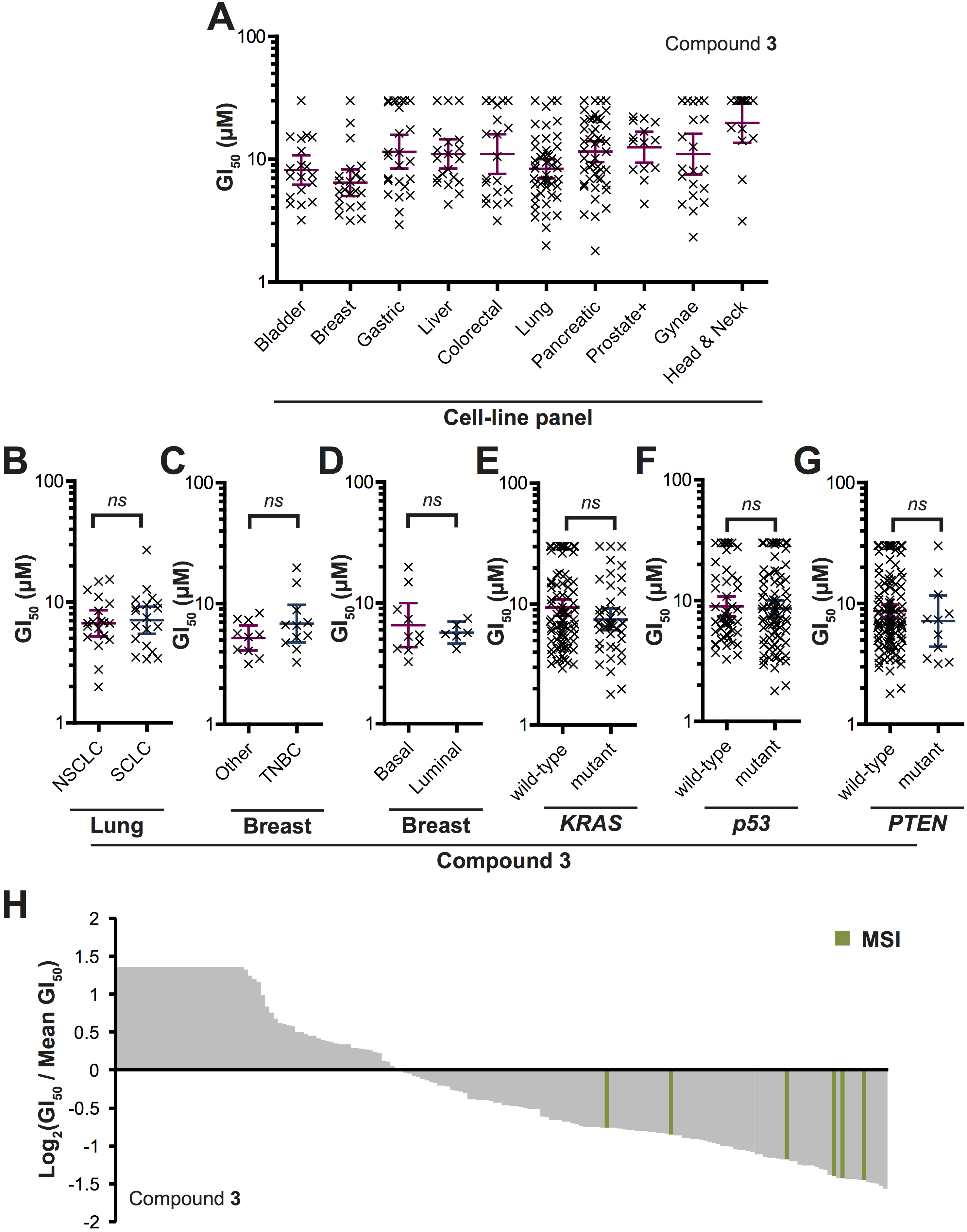

Supplement: S3 Fig — Scatter plots show cell-line sensitivities to treatment with 3, broadly grouped by tissue of origin (A), tissue sub-group (B-D) or oncogene mutation status (E-G). Waterfall plot (H) shows all cell-line sensitivities to 3, with those confirmed to be MSI highlighted in green. (TIF) [file pone.0179278.s003.tif]

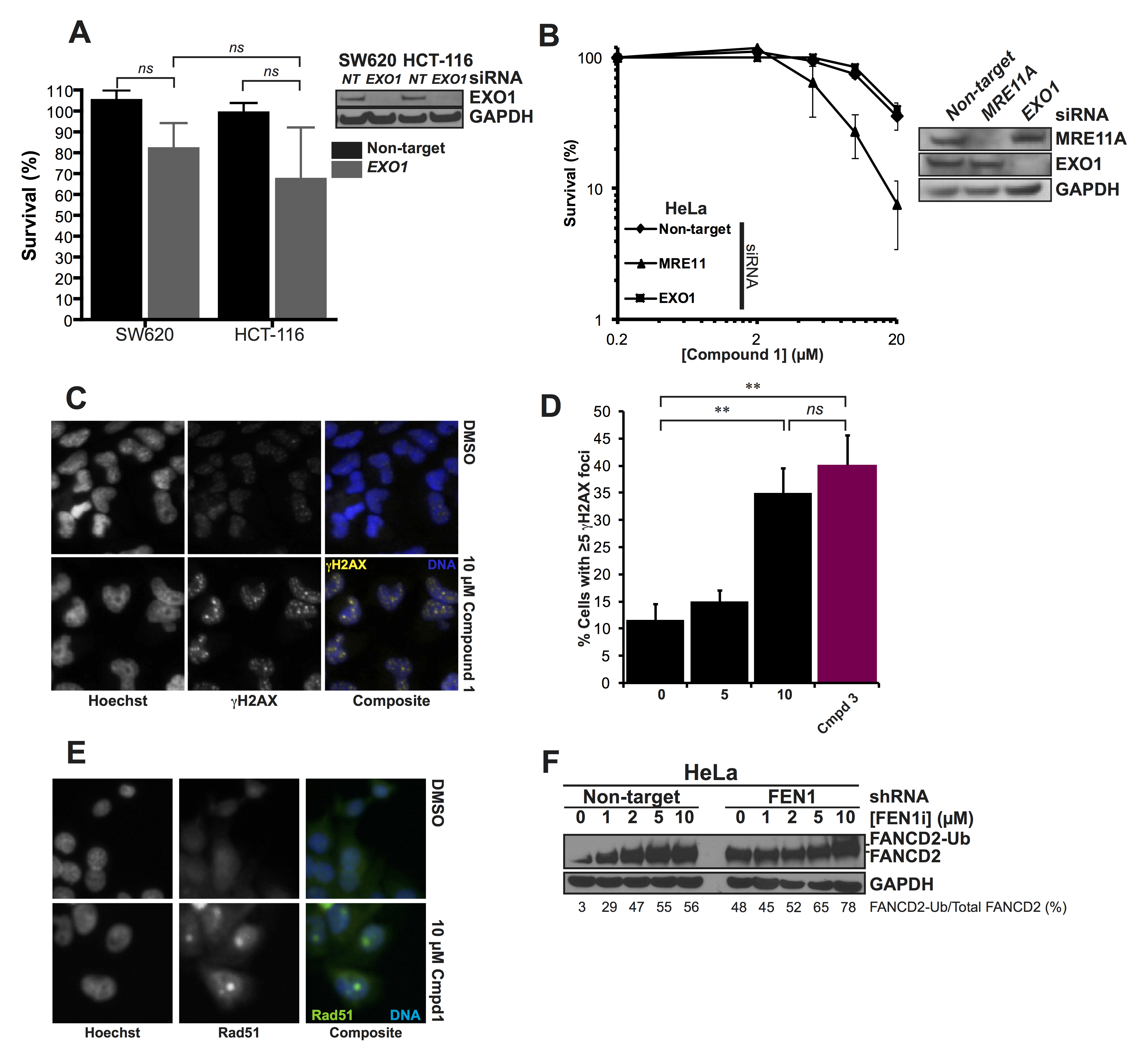

Supplement: S4 Fig — A. Clonogenic survival of SW620 and HCT-116 cells treated with siRNA against EXO1. B. Clonogeneic survival of HeLa cells disrupted for EXO1 and MRE11 following treatment with 1. C. Example of γH2AX foci induced by 1 or olaparib. D. Dose dependent increase in cells with at least 5 γH2AX foci. Data is a quantification from at least 500 cells. E. Example of RAD51 foci induced by 1 or 3. G. Activation of the Fanconi anemia pathway in cells disrupted for FEN1 compared to a non-target control. (TIF) [file pone.0179278.s004.tif]

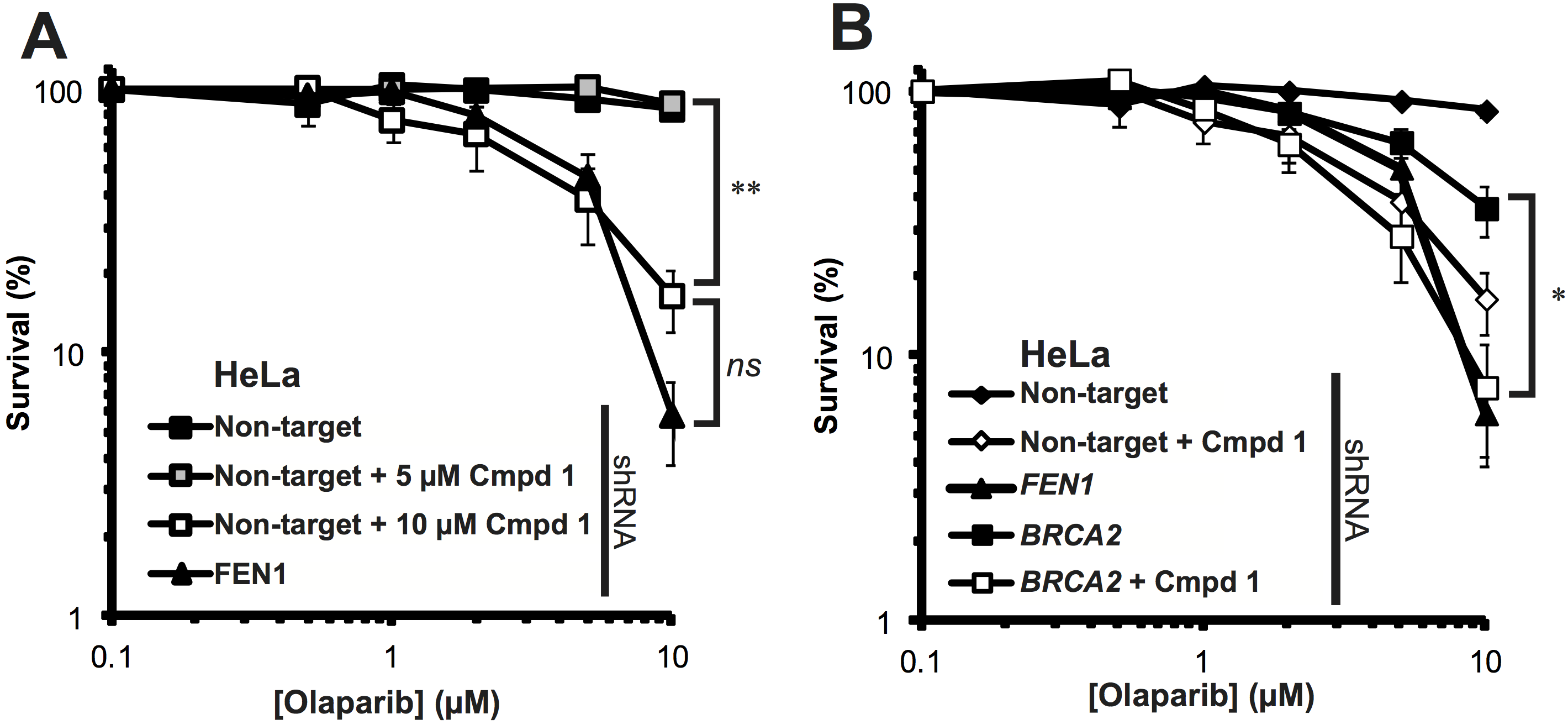

Supplement: S5 Fig — A. Effect treatment with 5 μM and 10 μM 1 has on sensitivity to olaparib in cells with wild-type levels of FEN1. B. Epistasis analysis of FEN1 inhibition and BRCA2 depletion following exposure to olaparib. (TIF) [file pone.0179278.s005.tif]

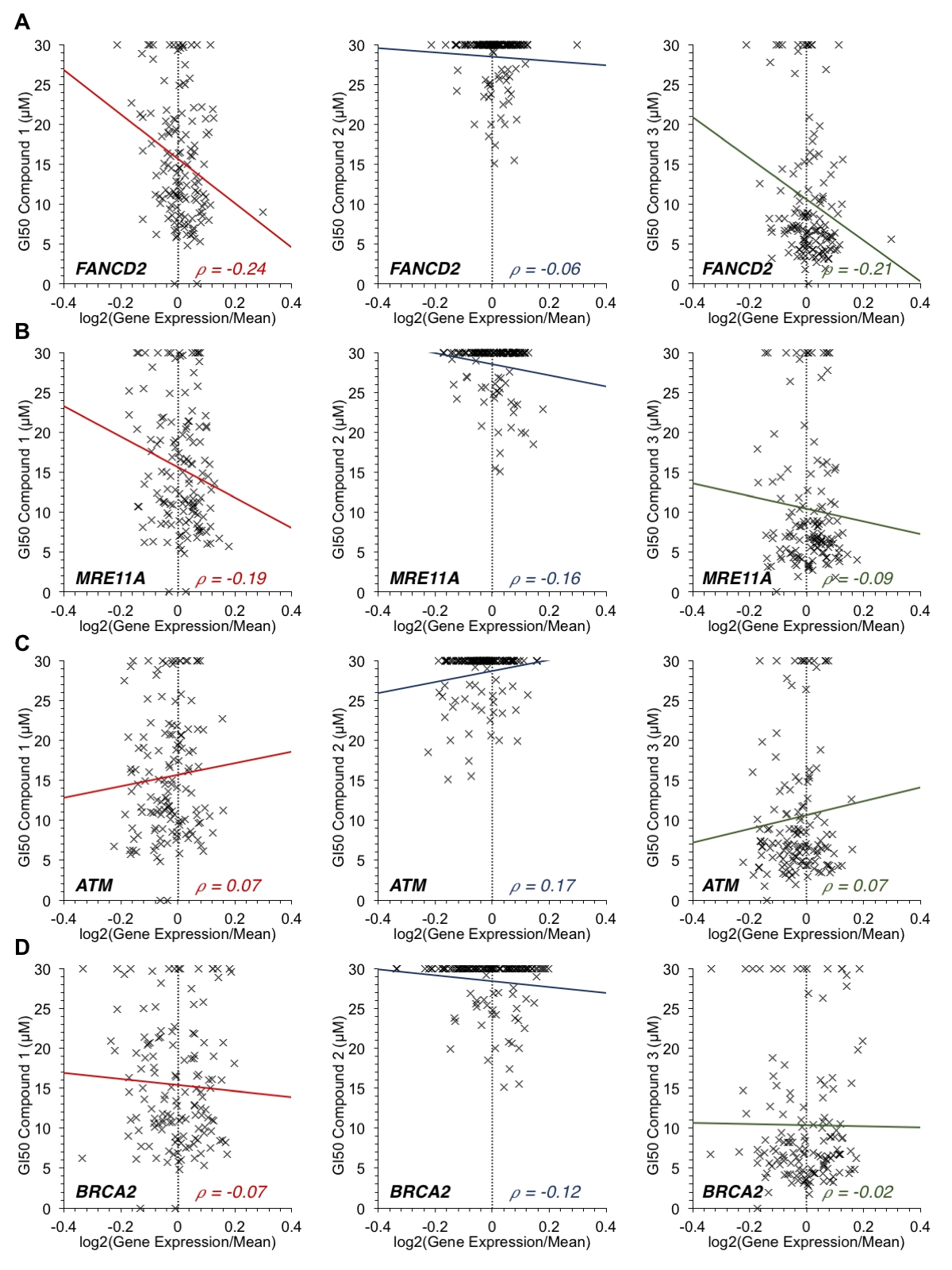

Supplement: S6 Fig — ρ denotes the Pearson’s correlation coefficient. (TIF) [file pone.0179278.s006.tif]

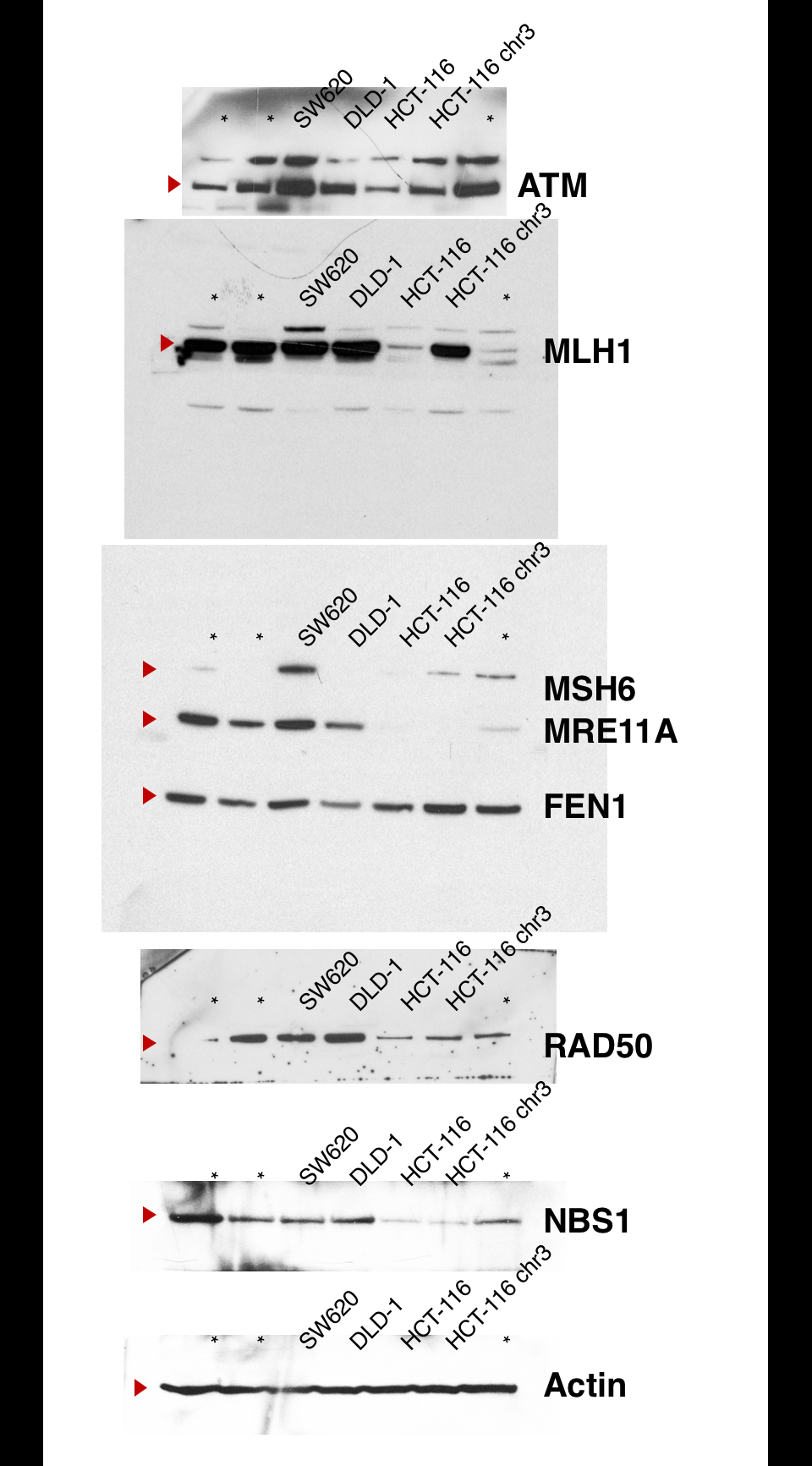

Supplement: S7 Fig — (TIF) [file pone.0179278.s007.tif]

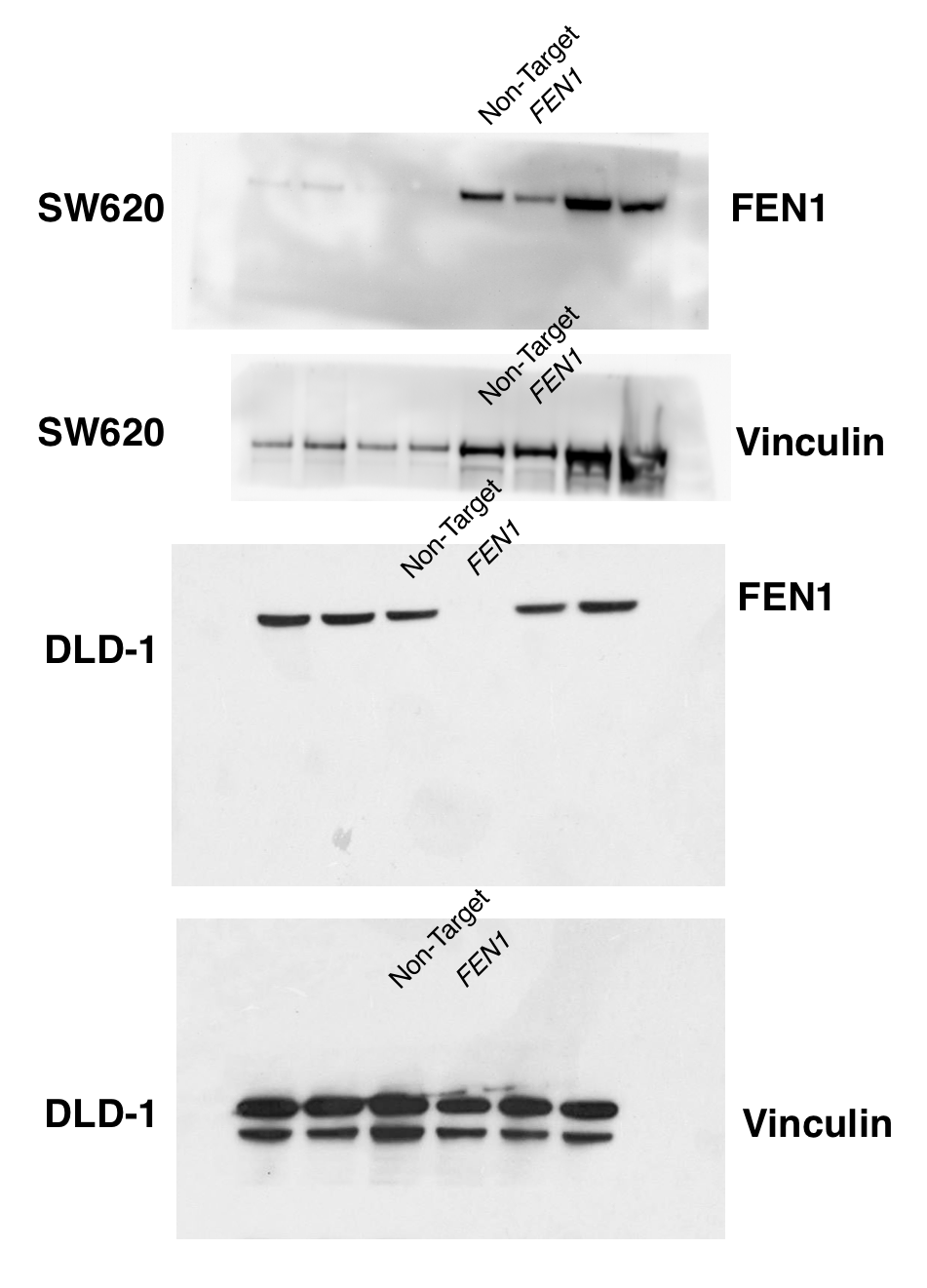

Supplement: S8 Fig — (TIF) [file pone.0179278.s008.tif]

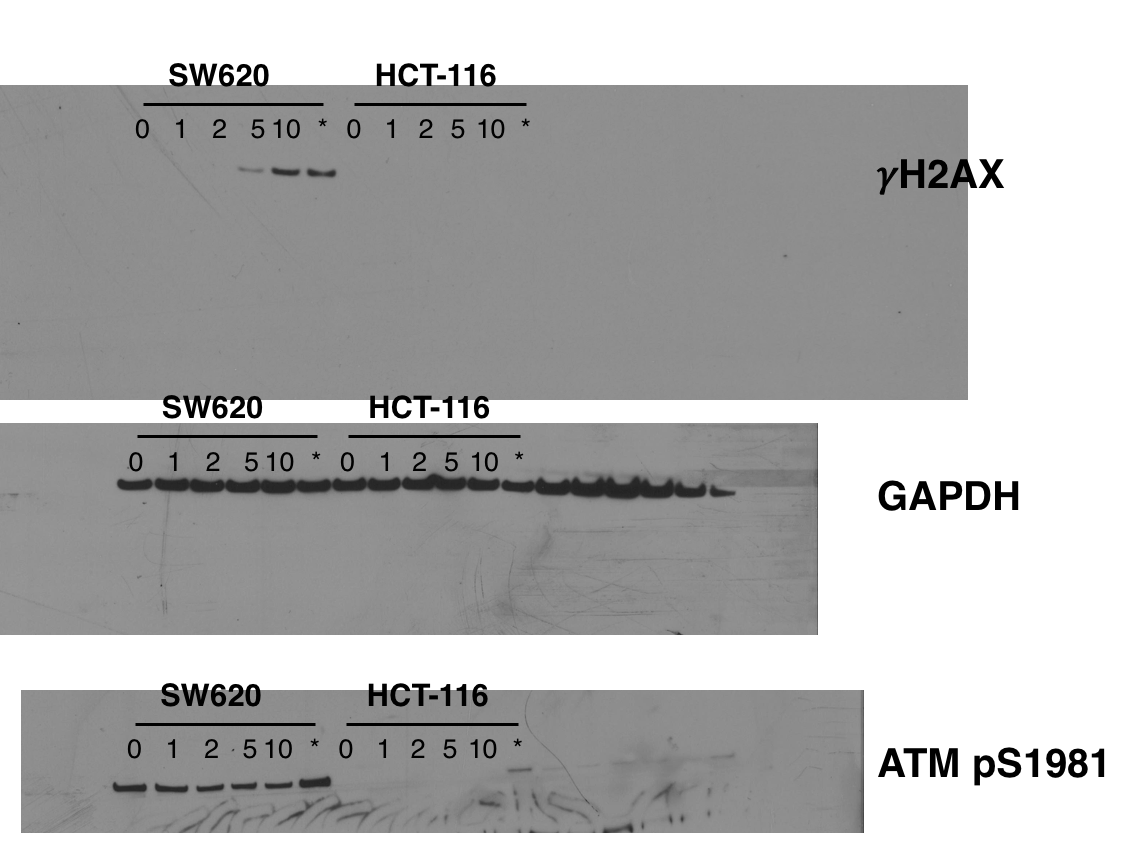

Supplement: S9 Fig — (TIF) [file pone.0179278.s009.tif]

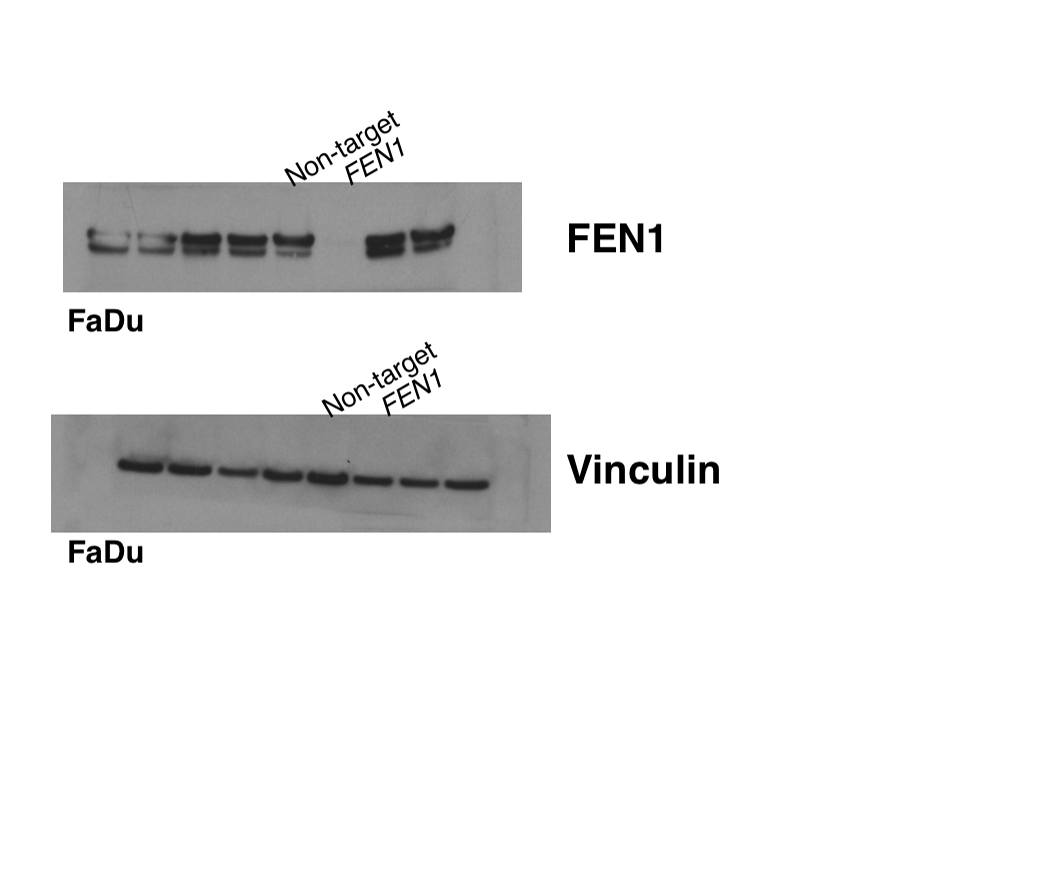

Supplement: S10 Fig — (TIF) [file pone.0179278.s010.tif]

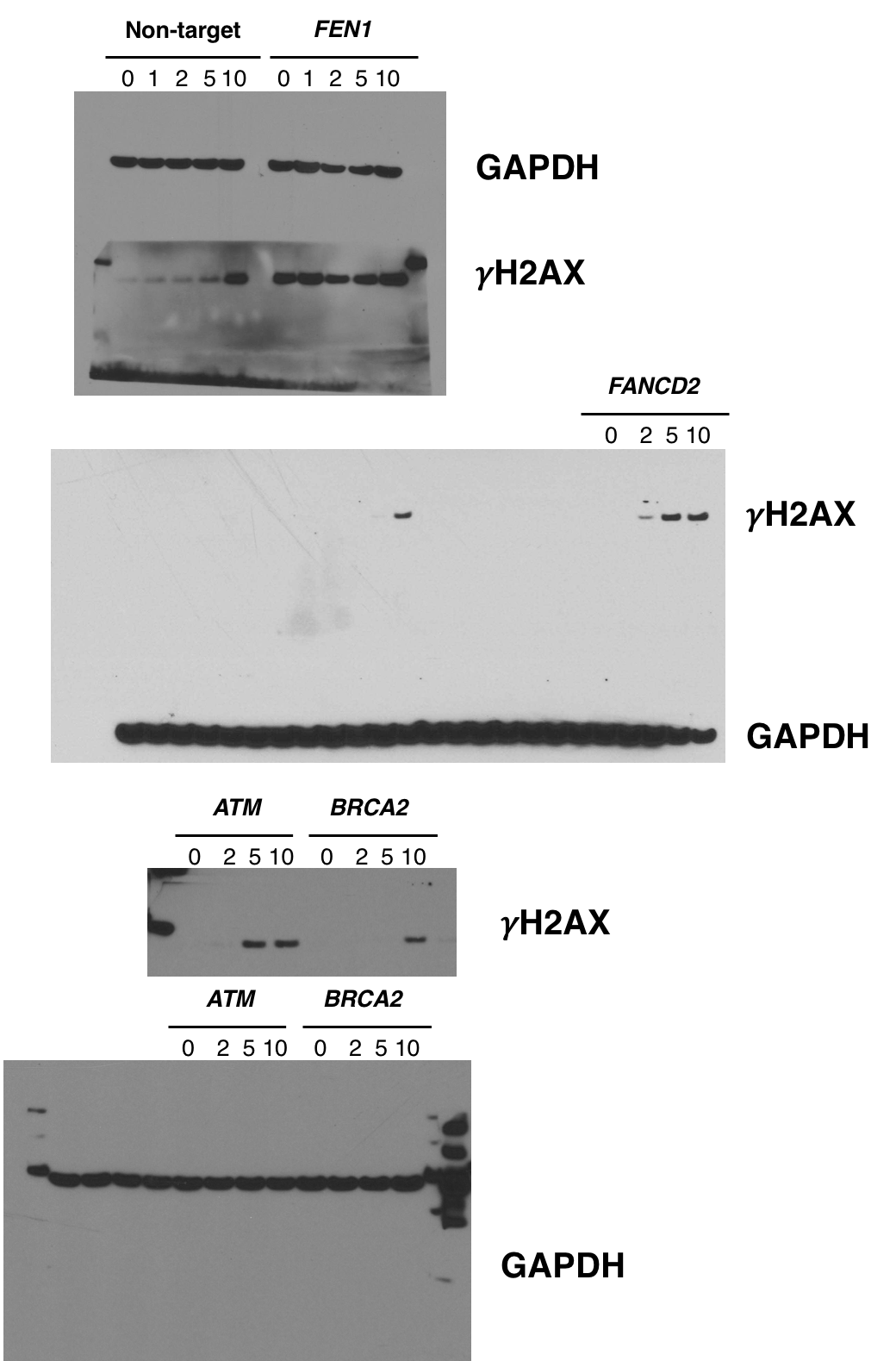

Supplement: S11 Fig — (TIF) [file pone.0179278.s011.tif]

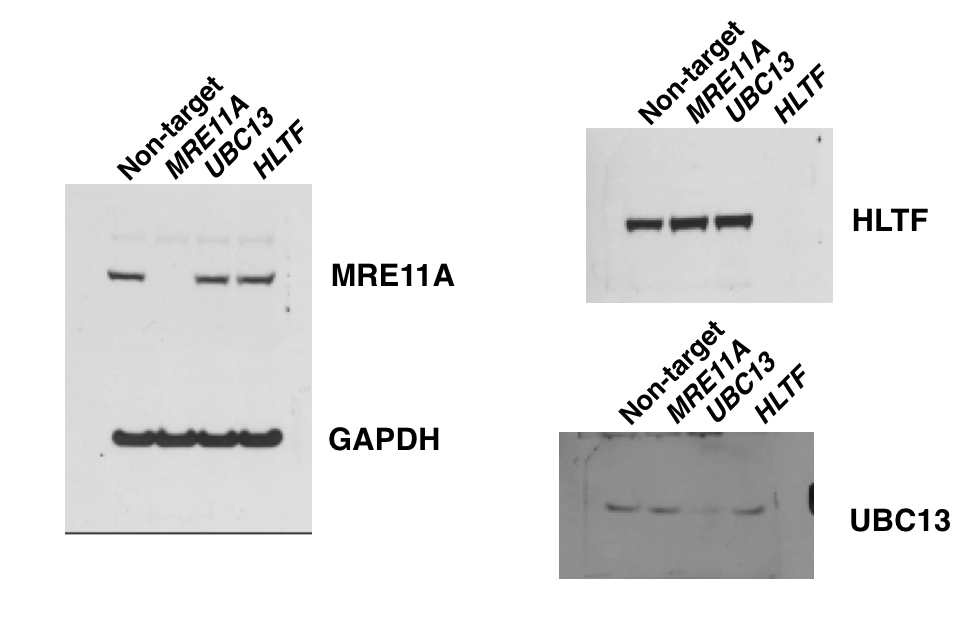

Supplement: S12 Fig — (TIF) [file pone.0179278.s012.tif]

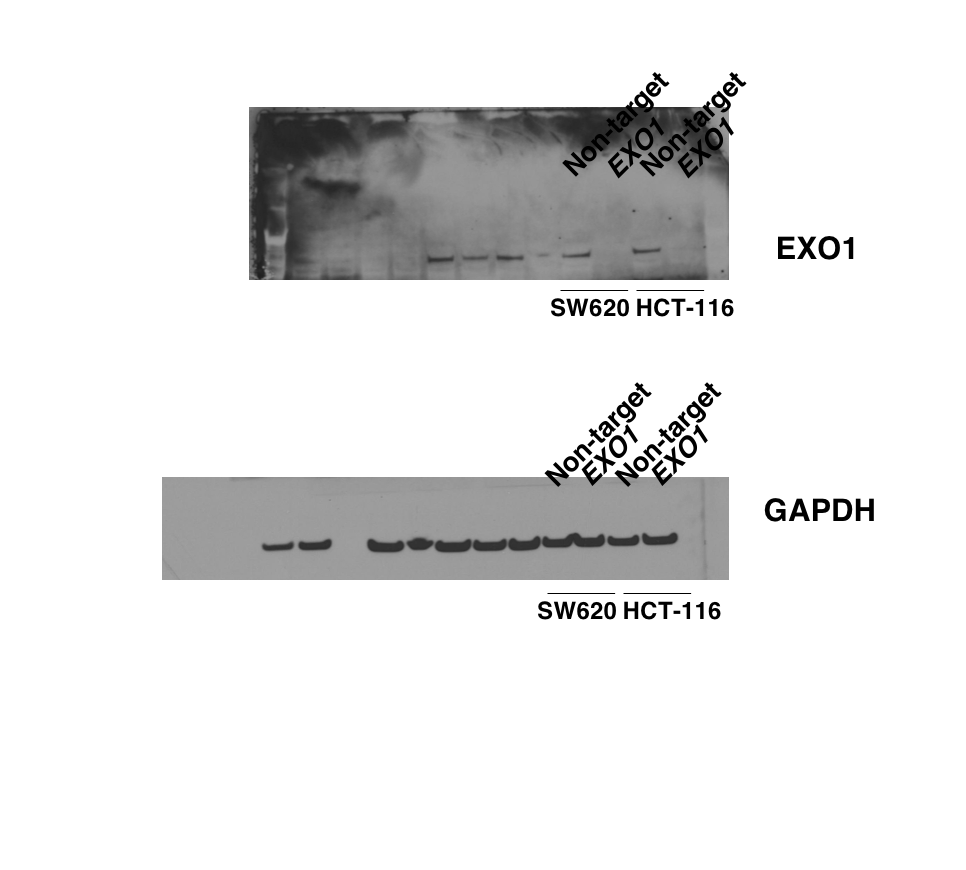

Supplement: S13 Fig — (TIF) [file pone.0179278.s013.tif]

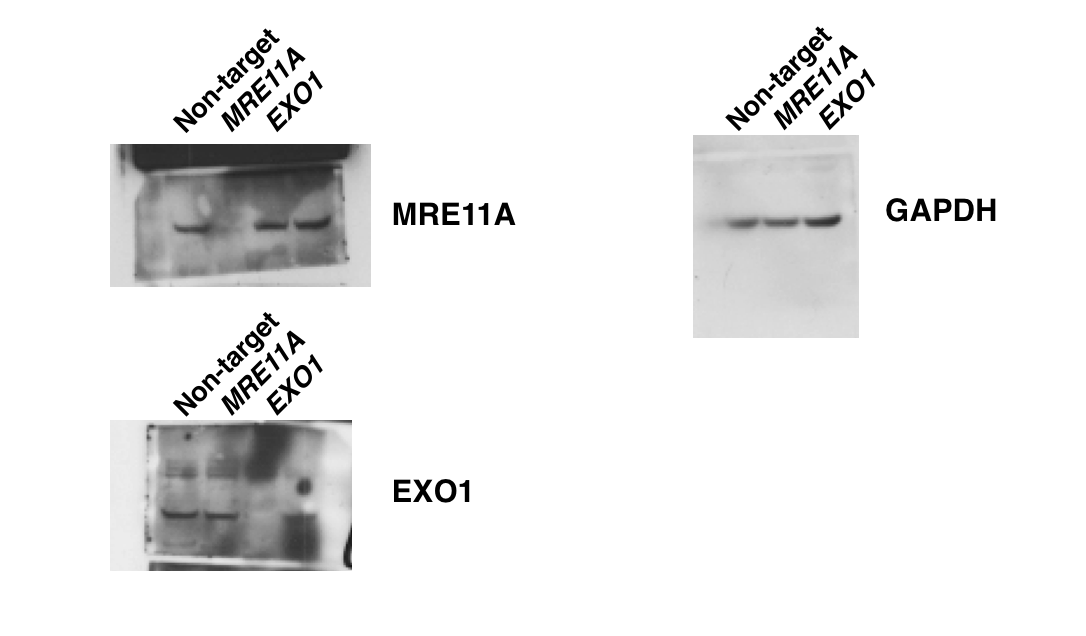

Supplement: S14 Fig — (TIF) [file pone.0179278.s014.tif]

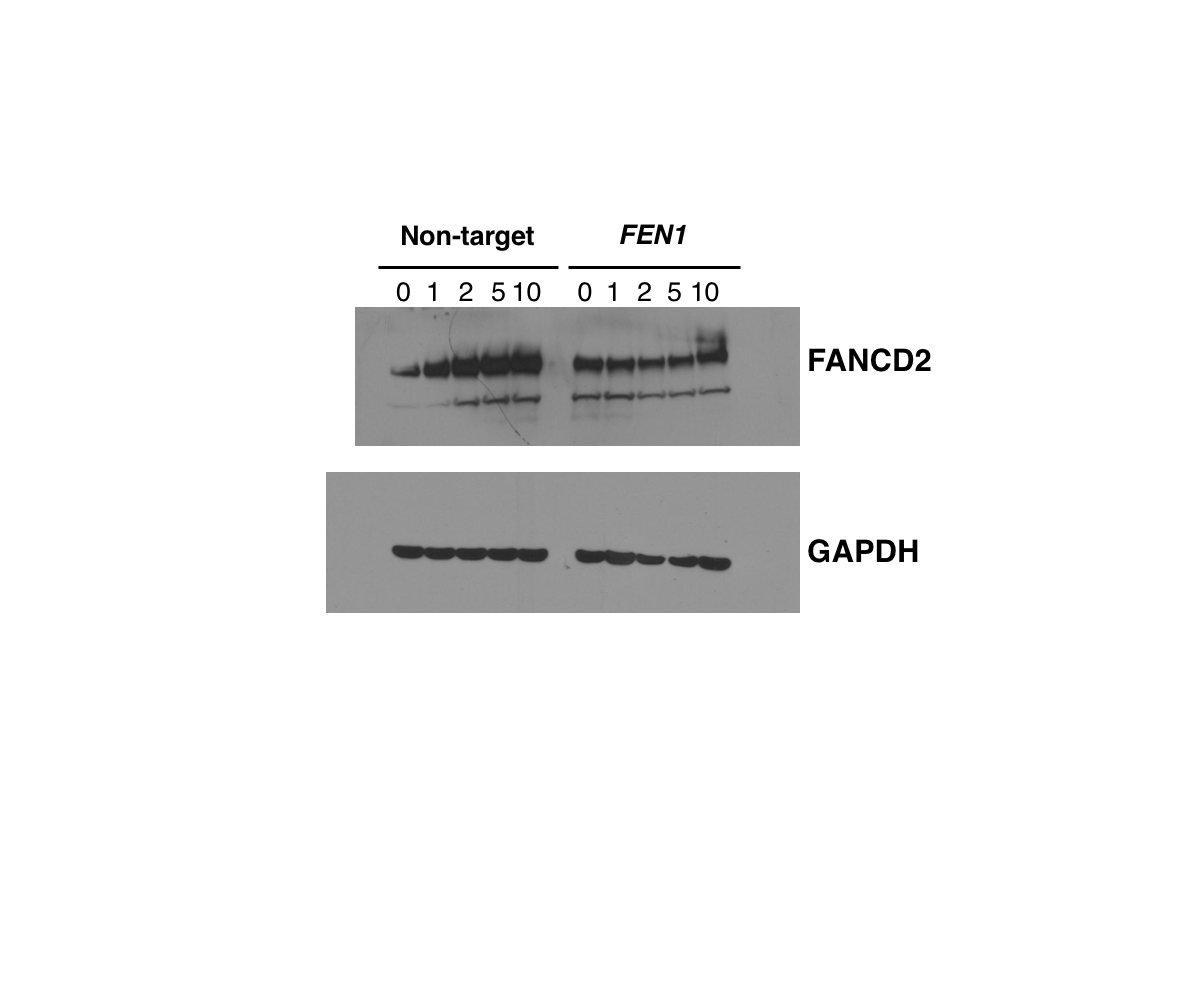

Supplement: S15 Fig — (TIF) [file pone.0179278.s015.tif]
