# Supplementary material for: Small molecule inhibitors uncover synthetic genetic interactions of human flap endonuclease 1 (FEN1) with DNA damage response genes
Source: PLoS One. 2017 Jun 19;12(6):e0179278. doi: 10.1371/journal.pone.0179278 (PMC5476263; doi:10.1371/journal.pone.0179278)
Supplement: S1 Table — (DOCX) [file pone.0179278.s016.docx]

| **Cell-Line** | **Tissue** | **Compound 1** | **Compound 2** | **Compound 3** |
| --- | --- | --- | --- | --- |
| 1A6 | Bladder | 10.83 | >30 | 6.451 |
| 5637 | Bladder | 9.03 | >30 | 5.602 |
| 647-V | Bladder | 5.324 | 15.54 | 3.198 |
| 97-7 | Bladder | 24.75 | >30 | 14.76 |
| BFTC-905 | Bladder | 11.29 | >30 | 4.911 |
| HT-1197 | Bladder | 16.01 | >30 | 7.291 |
| HT-1376 | Bladder | 10.93 | >30 | 8.697 |
| J82 | Bladder | 16.39 | >30 | 8.917 |
| KU-19-19 | Bladder | 16.03 | >30 | 7.174 |
| MGH-U3 | Bladder | 18.11 | >30 | 15.12 |
| RT112/84 | Bladder | 20.74 | >30 | 15.61 |
| RT4 | Bladder | 29.65 | >30 | >30 |
| SCaBER | Bladder | 13.61 | >30 | 7.445 |
| SW 780 | Bladder | 20.63 | >30 | 15.27 |
| SW-1710 | Bladder | 17.64 | >30 | 11.7 |
| T24 | Bladder | 7.498 | >30 | 4.33 |
| TCCSUP | Bladder | 12.93 | >30 | 8.418 |
| UM-UC-3 | Bladder | 10.54 | >30 | 4.234 |
| VM-CUB1 | Bladder | 9.602 | >30 | 4.481 |
| BT-20 | Breast | 20.73 | >30 | 14.87 |
| BT-549 | Breast | 12.42 | >30 | 5.433 |
| CAMA-1 | Breast | 10.71 | 25.98 | 3.17 |
| HCC1187 | Breast | 6.74 | 18.52 | 4.75 |
| HCC1395 | Breast | >30 | >30 | 19.8 |
| HCC1419 | Breast | 6.283 | 26.88 | 4.152 |
| HCC1569 | Breast | 16.35 | >30 | 7.322 |
| HCC1806 | Breast | 9.43 | 23.47 | 6.667 |
| HCC1937 | Breast | 19.02 | >30 | 8.813 |
| HCC1954 | Breast | 5.718 | 22.86 | 4.067 |
| JIMT-1 | Breast | 6.984 | 24.26 | 3.502 |
| MCF7 | Breast | 19.06 | >30 | 5.294 |
| MCF7/mdr+ | Breast | 27.08 | >30 | 8.347 |
| MDA-MB-157 | Breast | 15.05 | >30 | 5.271 |
| MDA-MB-231 | Breast | 14.08 | >30 | 6.982 |
| MDA-MB-436 | Breast | 10.9 | >30 | 4.184 |
| MDA-MB-453 | Breast | 12.84 | >30 | 5.537 |
| MDA-MB-468 | Breast | 8.151 | 24.19 | 3.269 |
| SK-BR-3 | Breast | 11.07 | 25.18 | 5.656 |
| SUM52PE | Breast | 14.54 | >30 | 6.582 |
| T-47D | Breast | >30 | >30 | >30 |
| ZR-75-1 | Breast | 15.07 | >30 | 7.447 |
| AZ-521 | Colorectal | 16.14 | 26.17 | 10.52 |
| C99 | Colorectal | >30 | >30 | >30 |
| CC20 | Colorectal | 22.43 | >30 | 6.913 |
| CCK-81 | Colorectal | >30 | >30 | >30 |
| COLO 205 | Colorectal | 29.6 | >30 | >30 |
| COLO 320DM | Colorectal | 11.65 | 23.11 | 5.411 |
| HCA-7 | Colorectal | 18.04 | >30 | 16.02 |
| HCT 116 | Colorectal | 8.986 | 26.96 | 4.463 |
| HCT-15 | Colorectal | 8.885 | >30 | 3.157 |
| HCT-8 | Colorectal | 8.505 | 28.94 | 4.803 |
| HRA-19 | Colorectal | >30 | >30 | >30 |
| HT-29 | Colorectal | 28.87 | >30 | 27.73 |
| LS 180 | Colorectal | 25.21 | >30 | 17.94 |
| LoVo | Colorectal | 10.98 | >30 | 6.497 |
| RKO | Colorectal | 10.68 | 29.15 | 4.284 |
| SK-CO-1 | Colorectal | 27.48 | >30 | 16.04 |
| SW403 | Colorectal | >30 | >30 | >30 |
| SW48 | Colorectal | 11.1 | >30 | 4.358 |
| SW480 | Colorectal | 11 | >30 | 6.694 |
| SW620 | Colorectal | 7.767 | 23.37 | 5.676 |
| SW948 | Colorectal | 18.12 | >30 | 20.9 |
| 23132/87 | Gastric | 5.965 | 20.17 | 3.708 |
| AGS | Gastric | 7.64 | 15.07 | 6.604 |
| GTL16 | Gastric | >30 | >30 | >30 |
| HGC-27 | Gastric | 9.926 | 27.59 | 4.91 |
| Hs 746T | Gastric | >30 | >30 | >30 |
| IM95m | Gastric | >30 | >30 | >30 |
| KATO III | Gastric | 7.541 | 22.52 | 6.726 |
| MKN1 | Gastric | 12.56 | >30 | 10.69 |
| MKN74 | Gastric | >30 | >30 | 29.21 |
| NCI-N87 | Gastric | 8.496 | 20.63 | 6.002 |
| NUGC-3 | Gastric | 14.9 | >30 | 11.36 |
| NUGC-4 | Gastric | 29.26 | >30 | >30 |
| OCUM-1 | Gastric | >30 | >30 | >30 |
| OE19 | Gastric | 17.94 | >30 | 17.48 |
| OE33 | Gastric | 8.343 | >30 | 5.083 |
| PAMC82 | Gastric | 22.02 | >30 | 9.612 |
| SNU-1 | Gastric | 8.698 | 17.44 | 5.159 |
| SNU-16 | Gastric | 25.78 | >30 | 16.26 |
| SNU-216 | Gastric | 18.76 | >30 | >30 |
| SNU-484 | Gastric | 11.75 | >30 | 11.41 |
| SNU-5 | Gastric | 5.853 | 24.76 | 2.937 |
| SNU-601 | Gastric | 11.63 | 25.02 | 8.91 |
| SNU-620 | Gastric | 21.76 | >30 | 26.33 |
| SNU-638 | Gastric | 14.02 | >30 | 6.942 |
| SNU-668 | Gastric | 18.52 | >30 | 6.95 |
| AN3 CA | Gynae | 15.28 |  | 2.325 |
| COV362 | Gynae | >30 |  | >30 |
| EFO-27 | Gynae | 20.94 |  | 5.784 |
| EN | Gynae | 22.83 |  | 29.57 |
| HEC-1-A | Gynae | 22.62 |  | 8.242 |
| IGR-OV1 | Gynae | 25.12 |  | 6.274 |
| KLE | Gynae | >30 |  | >30 |
| KURAMOCHI | Gynae | >30 |  | 12.63 |
| MFE-280 | Gynae | >30 |  | >30 |
| MFE-296 | Gynae | 27.24 |  | 3.776 |
| NIH:OVCAR-3 | Gynae | 14.55 | >30 | 5.054 |
| OAW28 | Gynae | >30 |  | >30 |
| OAW42 | Gynae | >30 |  | >30 |
| OVCAR-4 | Gynae | 16.09 |  | 4.434 |
| OVCAR-5 | Gynae | >30 |  | 15.24 |
| RL95-2 | Gynae | 21.45 |  | 21.25 |
| RMG-I | Gynae | 11.84 |  | 8.057 |
| SK-OV-3 | Gynae | 23.96 |  | 7.266 |
| SNG-M | Gynae | 20.35 |  | 4.288 |
| TOV-112D | Gynae | 25.39 |  | 20.94 |
| TOV-21G | Gynae | 24.63 |  | 8.359 |
| BEL7404 | Liver | 10.8 | >30 | 5.233 |
| BEL7405 | Liver | 18.34 | >30 | 11.05 |
| HCCC9810 | Liver | 24.6 | >30 | >30 |
| HLE | Liver | 16.54 | >30 | 6.175 |
| HLF | Liver | 13.61 | >30 | 8.263 |
| Hep 3B | Liver | >30 | >30 | >30 |
| Hep G2 | Liver | 9.87 | 25.57 | 4.291 |
| HuH-7 | Liver | 21.85 | >30 | 10.06 |
| MHCC97-L | Liver | 24.92 | >30 | >30 |
| QGY7703 | Liver | 17.36 | >30 | 7.59 |
| SK-HEP-1 | Liver | 16.7 | 26.77 | 6.527 |
| SMMC-7721 | Liver | 15.66 | >30 | 10.17 |
| SNU-354 | Liver | >30 | >30 | 16.54 |
| SNU-368 | Liver | 26.5 | >30 | 12.2 |
| SNU-398 | Liver | 18.74 | >30 | 6.47 |
| SNU-449 | Liver | 24.88 | >30 | 11.84 |
| SNU-739 | Liver | 17.76 | >30 | 6.977 |
| SNU-761 | Liver | 28.13 | >30 | 14.38 |
| SNU-878 | Liver | 20.38 | >30 | 7.156 |
| SNU-886 | Liver | 25.51 | >30 | 13.83 |
| huH-1 | Liver | >30 | >30 | >30 |
| A549 | Lung | 11.3 | >30 | 6.97 |
| Calu-3 | Lung | 11.59 | >30 | 15.38 |
| Calu-6 | Lung |  | 23.82 | 1.983 |
| DMS 114 | Lung | 7.093 | 26.05 | 3.428 |
| HX147 | Lung | 21.99 | >30 | 9.686 |
| NCI-H1299 | Lung | 16.63 | >30 | 6.697 |
| NCI-H1437 | Lung | 10.64 | >30 | 6.664 |
| NCI-H1793 | Lung | 12.84 | >30 | 8.44 |
| NCI-H1975 | Lung | 19.66 | >30 | 14.76 |
| NCI-H2085 | Lung | 7.708 | >30 | 6.041 |
| NCI-H2126 | Lung | 22.71 | >30 | 12.62 |
| NCI-H2291 | Lung | 6.259 | >30 | 6.768 |
| NCI-H23 | Lung | 4.854 | 26.89 | 2.765 |
| NCI-H322 | Lung | 22.09 | >30 | 11.03 |
| NCI-H358 | Lung | 7.996 | >30 | 4.364 |
| NCI-H460 | Lung |  | >30 | 6.879 |
| NCI-H460 dnp53 | Lung |  | 29.6 | 5.107 |
| NCI-H522 | Lung | 11.24 | >30 | 6.296 |
| NCI-H526 | Lung | 6.142 | 19.94 | 3.859 |
| NCI-H838 | Lung | 8.222 | >30 | 4.885 |
| PC-9 | Lung | 10.53 | 29.87 | 5.271 |
| Calu-1 | Lung | 17.4 | >30 | 12.73 |
| EBC-1 | Lung | 6.217 | 20.81 | 3.365 |
| HARA | Lung | 13.46 | >30 | 7.44 |
| HCC-15 | Lung | 9.702 | >30 | 4.328 |
| HCC-95 | Lung | 18.66 | >30 | 10.8 |
| LK-2 | Lung | 11.73 | >30 | 6.301 |
| LUDLU-1 | Lung | >30 | >30 | 26.91 |
| NCI-H1703 | Lung | 11.3 | >30 | 9.758 |
| NCI-H1869 | Lung | 6.691 | >30 | 6.257 |
| NCI-H2170 | Lung | 12.28 | >30 | 8.942 |
| NCI-H226 | Lung | 19.4 | >30 | 8.501 |
| NCI-H2286 | Lung | 6.789 | 23.8 | 3.49 |
| NCI-H520 | Lung | 10.11 | >30 | 5.945 |
| NCI-H596 | Lung | >30 | >30 | >30 |
| NCI-H647 | Lung | 11.8 | >30 | 8.9 |
| RERF-LC-AI | Lung | 20.44 | >30 | >30 |
| RERF-LC-Sq1 | Lung | 10.69 | >30 | 4.954 |
| SK-MES-1 | Lung | 18.98 | >30 | 9.462 |
| SW 900 | Lung | 14.53 | >30 | 10.42 |
| AsPC-1 | Pancreatic | >30 | >30 | >30 |
| BxPC-3 | Pancreatic | 20.43 | 25.43 | 10.16 |
| CFPAC-1 | Pancreatic | 20.87 | >30 | 7.023 |
| Capan-1 | Pancreatic | 21.4 | >30 | 16.48 |
| Capan-2 | Pancreatic | 14.47 | >30 | 8.956 |
| HPAC | Pancreatic | 25.02 | >30 | 18.8 |
| HPAF-II | Pancreatic | 25.22 | >30 | 14.28 |
| Hs 766T | Pancreatic | >30 | >30 | >30 |
| HuP-T3 | Pancreatic | 6.011 | 19.97 | 1.794 |
| HuP-T4 | Pancreatic | >30 | >30 | 23.1 |
| KP-4 | Pancreatic | 11.55 | >30 | 5.862 |
| MIA PaCa-2 | Pancreatic | 10.16 | 20.03 | 3.968 |
| PANC-1 | Pancreatic | 21.35 | >30 | 8.192 |
| PANC-89 | Pancreatic | 17.58 | >30 | 5.624 |
| Panc 02.03 | Pancreatic | 11.99 | >30 | 4.724 |
| Panc 03.27 | Pancreatic | 14.79 | >30 | 6.944 |
| Panc 04.03 | Pancreatic | 13.93 | >30 | 6.932 |
| Panc 08.13 | Pancreatic | 13.48 | >30 | 6.143 |
| Panc 10.05 | Pancreatic | 8.222 | >30 | 3.409 |
| QGP-1 | Pancreatic | 8.659 | >30 | 3.547 |
| SU.86.86 | Pancreatic | 21.25 | >30 | 8.245 |
| SW 1990 | Pancreatic | 17.59 | >30 | 7.669 |
| T3M-4 | Pancreatic | 13.92 | >30 | 6.259 |
| YAPC | Pancreatic | 21.42 | >30 | 8.706 |
| 22Rv1 | Prostate | 22.16 | >30 | 13.66 |
| A2058 | Prostate | 8.44 | 25.7 | 4.325 |
| DU 145 | Prostate | 15 | >30 | 6.755 |
| LNCAP-CasRes (In house) | Prostate | 21.54 | >30 | 14 |
| LNCaP clone FGC | Prostate | 16.87 | >30 | 8.24 |
| PC-3 | Prostate | 20.68 | >30 | 8.515 |
| PNT1A | Prostate | 20.04 | >30 | 14.34 |

S1 Table. High-throughput screen for genetic backgrounds sensitive to *N*-hydroxyurea series inhibitors of FEN1 – raw GI_50_ values
