# Supplementary material for: Small molecule inhibitors uncover synthetic genetic interactions of human flap endonuclease 1 (FEN1) with DNA damage response genes
Source: PLoS One. 2017 Jun 19;12(6):e0179278. doi: 10.1371/journal.pone.0179278 (PMC5476263; doi:10.1371/journal.pone.0179278)
Supplement: S2 Table — * Strain kindly donated by the Boone Lab. # Strains purchased from Open Biosystems. (DOCX) [file pone.0179278.s017.docx]

| **Strain** | **Genotype** | **Ref** |
| --- | --- | --- |
| BY4741 | *MAT*a *his3Δ1 leu2Δ0 lys2Δ0 ura3Δ0* | Lab Stock |
| Y8205 | MATα *his3Δ1 leu2Δ0 met15Δ0 ura3Δ0can1Δ::STE2pr-Sp_his5*  *lyp1Δ::STE3pr-LEU2* | * |
| 6743 | BY4741 with *pso2∷kanMX6* | # |
| 6240 | BY4741 with *msh2∷kanMX6* | # |
| 4963 | BY4741 with *rad27∷kanMX6* | # |
| TWY4 | Y8205 with *pso2∷kanMX6* | [89] |
| TWY5 | Y8205 *pso2::natMX6 msh2::hphMX4* | [89] |
| TWY7 | *MAT*a double mutant spore from Y8205 *pso2::hphMX4* x BY4741 *msh2::kanMX6* | [89] |
| TWY74 | *MAT*a double mutant spore from Y8205 *pso2::hphMX4* x BY4741 *rad27::kanMX6* | This study |
| TWY75 | *MAT*a triple mutant spore from Y8205 *pso2::natMX6 msh2::hphMX4* x BY4741 *rad27::kanMX6* | This study |

S2 Table. Yeast strains used in this study. * Strain kindly donated by the Boone Lab. ^#^ Strains purchased from Open Biosystems.
